# Controlling genetic heterogeneity in gene-edited hematopoietic stem cells by single-cell expansion

**DOI:** 10.1016/j.stem.2023.06.002

**Published:** 2023-07-06

**Authors:** Hans Jiro Becker, Reiko Ishida, Adam C. Wilkinson, Takaharu Kimura, Michelle Sue Jann Lee, Cevayir Coban, Yasunori Ota, Yosuke Tanaka, Meike Roskamp, Tsubasa Sano, Arinobu Tojo, David G. Kent, Satoshi Yamazaki

**Affiliations:** 1 Laboratory for Stem Cell Therapy, Faculty of Medicine, Tsukuba University, Tsukuba 305-8577, Japan; 2 Division of Stem Cell Biology, Center for Stem Cell Therapy, The Institute of Medical Science, The University of Tokyo, Tokyo 108-8639, Japan; 3 MRC Weatherall Institute of Molecular Medicine, Radcliffe Department of Medicine, University of Oxford, Oxford OX3 9DS, UK; 4 Division of Malaria Immunology and International Vaccine Design Center, The Institute of Medical Science, The University of Tokyo, Tokyo 108-8639, Japan; 5 Department of Pathology, Research Hospital, The Institute of Medical Science, The University of Tokyo, Tokyo 108-8639, Japan; 6 International Research Center for Medical Sciences, Kumamoto University, Kumamoto City 860-0811, Japan; 7 Pharma Solutions, Nutrition & Health, BASF SE, Carl-Bosch-Strasse 38, 67056 Ludwigshafen am Rhein, Germany; 8 Pharma Solutions, Nutrition & Health, BASF Japan Ltd, Tokyo 103-0022, Japan; 9 Tokyo Medical and Dental University, Tokyo 113-8510, Japan; 10 York Biomedical Research Institute, Department of Biology, University of York, Wentworth Way, York YO10 5DD, UK

**Keywords:** hematopoietic stem cell, *ex vivo* expansion, clonal expansion, chemically defined culture, stem cell culture, gene editing, CRISPR-Cas9, transplantation, regenerative medicine, gene therapy

## Abstract

Gene editing using engineered nucleases frequently produces unintended genetic lesions in hematopoietic stem cells (HSCs). Gene-edited HSC cultures thus contain heterogeneous populations, the majority of which either do not carry the desired edit or harbor unwanted mutations. In consequence, transplanting edited HSCs carries the risks of suboptimal efficiency and of unwanted mutations in the graft. Here, we present an approach for expanding gene-edited HSCs at clonal density, allowing for genetic profiling of individual clones before transplantation. We achieved this by developing a defined, polymer-based expansion system and identifying long-term expanding clones within the CD201^+^CD150^+^CD48^−^c-Kit^+^Sca-1^+^Lin^−^ population of precultured HSCs. Using the *Prkdc*^scid^ immunodeficiency model, we demonstrate that we can expand and profile edited HSC clones to check for desired and unintended modifications, including large deletions. Transplantation of *Prkdc-*corrected HSCs rescued the immunodeficient phenotype. Our *ex vivo* manipulation platform establishes a paradigm to control genetic heterogeneity in HSC gene editing and therapy.

## Highlights

• Gene editing frequently produces unwanted mutations in transplanted HSC grafts
• *Ex vivo* expanding HSCs are enriched in the CD201^+^CD150^+^CD48^−^KSL population
• A polymer-supplemented culture system enables single-cell expansion of edited HSCs
• Expanded HSC clones can be screened to exclude specific clones from transplantation

## Graphical abstract

**Figure:**
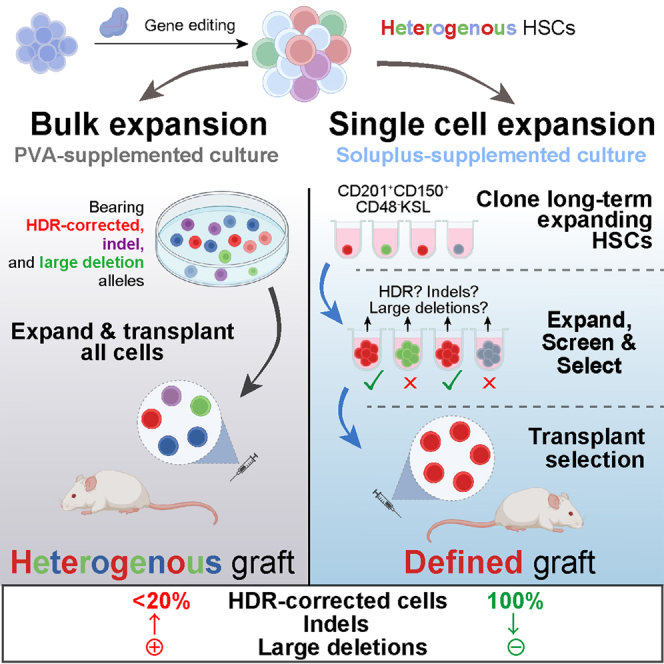

## Teaser

Becker and colleagues leverage a single-cell expansion system to assemble genetically defined grafts from heterogeneous gene-edited murine hematopoietic stem cell (HSC) cultures. The expansion platform presented here enables direct selection of *bona fide* HDR-corrected HSCs and the exclusion of clones bearing collateral modifications (indels, large deletions) from transplantation.

## Introduction

The rapid adoption of engineered nucleases has put hematopoietic stem cells (HSCs) at the center of gene editing applications. The ability to functionally interrogate genes by introducing or correcting mutations at precise loci has greatly advanced our understanding of HSC biology and has enabled curative approaches for genetic diseases. CRISPR-Cas9 currently represents the most widespread system for gene editing of the hematopoietic system.^1^ A target-specific guide RNA (gRNA) directs the Cas9 endonuclease to a genomic site of interest, where it induces a DNA double-strand break (DSB). The subsequent engagement of the cell-intrinsic DNA damage repair (DDR) machinery can be exploited to create targeted modifications in HSCs.^2^^,^^3^^,^^4^ Since mutagenic repair (e.g., non-homologous end joining [NHEJ]) takes precedence in primitive HSCs,^5^ a phenomenon closely tied to their dormant phenotype, random small insertions and deletions (indels) represent the most common editing outcome.^2^^,^^6^ Furthermore, a string of recent reports have uncovered previously underappreciated lesions, such as kilo- and megabase-scale deletions as well as chromothripsis, illustrating the potential risk of Cas9-based gene editing.^7^^,^^8^^,^^9^ In contrast, correction via templated repair (i.e., homology-directed repair [HDR]) among long-term (LT)-HSCs remains inefficient.^5^^,^^10^ Off-target mutations may also raise concerns about genotoxicity in edited cells.^11^ Together, these unwanted mutations may confound the effects of the targeted gene edit and represent incalculable risks in basic and translational research settings. Apart from gene editing, maintaining HSC self-renewal in LT cultures required by gene editing protocols remains challenging.^12^ Consequently, edited and bulk-expanded HSC cultures contain genetically and functionally heterogeneous populations and only include a low fraction of functional HSCs with the desired genetic modifications.

Expansion of single, *bona fide* HSCs would overcome this limitation by enabling direct profiling of on- and off-target editing outcomes, allowing for selective transplantation only of clones with a defined mutational pattern. However, current protocols do not allow for expansion of HSCs at clonal density to the extent necessary for transplantation. Clonal expansion technologies for embryonic stem cells (ESCs) and induced pluripotent stem cells (iPSCs) have been major drivers for advances in the biology and translational research of PSCs, yet the generation of functional HSCs from PSCs remains a major hurdle.

We recently reported on a serum-free, polyvinyl alcohol (PVA)-based HSC expansion protocol that permits up to 899-fold expansion of HSCs over a period of 4 weeks.^13^ Here, we use this protocol to show that bulk expansion produces a genetically heterogeneous graft with on- and off-target indels as well as large deletions (LDs). Addressing this issue, we present a system that supports single-cell expansion of edited HSCs and define a phenotype that assists in selecting precultured clones with LT expansion potential. We apply this system to a gene correction model of severe combined immunodeficiency (SCID), demonstrating the feasibility of single-cell expansion for sequence-based selection of edited HSC clones. Lastly, we use a human hemoglobin beta (HBB) editing model to underline the conceptual advantage of clonal expansion in a translational context.

## Results

### Gene-edited HSCs correct Prkdc^scid^ immunodeficiency but bear on- and off-target indels

**Figure 1:**
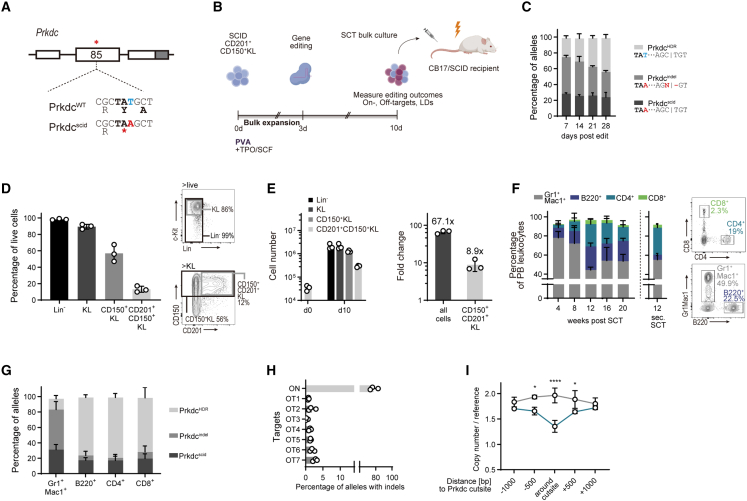
Autologous HSCT gene correction rescues the *Prkdc*^scid^ phenotype but introduces on-, off-target indels and large deletions (A) Genomic context of the *Prkdc*^scid^ mutation in exon 85. White boxes: exons, gray box, 3′ UTR. ^∗^ denotes location of *Prkdc*^scid^ mutation. (B) Experimental scheme of the gene editing and HSC expansion model. (C) Post-editing allele distribution at the *Prkdc* locus, assessed by inference of CRISPR edit (ICE) (n = 3 cultures). (D) Fractions of immunophenotypically defined HSPC populations within cultures on day 10 of culture, 7 days post-editing. Percentage of all live cells (n = 3 cultures). (E) Absolute cell numbers (left panel) and fold-change expansion (right panel) of cultured HSPCs, day 10 of culture. (F) Left: frequencies of peripheral blood (PB) leukocytes as percentage of all live leukocytes (n = 3 groups, 3–4 mice per group). Plot next to dashed line shows frequencies 12 weeks post-secondary SCT (n = 5 mice). Right: representative fluorescence-activated cell sorting (FACS) plots 20 weeks post-transplant. (G) Frequencies of *Prkdc* alleles in sorted PB cells 20 weeks post-SCT (n = 3 experiments, 3–4 mice per group). (H) On- and off-target (OT) activity of the *Prkdc*-specific gRNA, assessed with tracking of indels by decomposition (TIDE). The seven highest scoring off-target sites, as predicted by COSMID, were interrogated. See Table S1 for detailed information about the off-target sites. (I) Copy-number analysis of *Prkdc* probes against reference gene (n = 3). Two-way ANOVA with Sidak’s multiple comparison test. Error bars represent SD. ^∗^p < 0.05, ^∗∗∗∗^p < 0.0001.

The immunodeficient phenotype in CB17/SCID mice is caused by a T to A mutation in the *Prkdc* gene (*Prkdc*^scid^), p.Y4046X), leading to functional loss of its product, DNA-dependent protein kinase catalytic subunit (DNA-PKcs, Figure 1A).^14^ DNA-PKcs is indispensable for the resolution of DNA DSBs during V(D)J recombination, which is reflected in the absence of functional B and T cells in CB17/SCID mice.

To determine whether the *Prkdc*^scid^ phenotype can be corrected with gene-edited and bulk-expanded HSCs and to assess the levels of indels generated in the process, we designed a gene-editing protocol based on our previously established *ex vivo* HSC expansion platform (Figure 1B).^13^ CD201^+^CD150^+^c-Kit^+^Lin^−^ cells from CB17/SCID mice were cultured in PVA-based medium (PVA-HSC) for 3 days (Figure S1A). We included CD201 (endothelial protein C receptor, EPCR) in our isolation panel since the commonly employed marker stem cell antigen 1 (Sca-1) is known to be poorly expressed on hematopoietic cells of non-C57BL/6 mouse strains and because CD201 has shown to be a reliable marker in BALB/c mice, from which the CB17/SCID strain is derived.^15^ Cas9 ribonucleoprotein (RNP) complexes and a corrective HDR template were delivered into HSCs 3 days after isolation. 1 week after editing, the majority of alleles contained indels (*Prkdc*^indel^, 48%), while 26% had incorporated the HDR donor sequence (*Prkdc*^HDR^, Figure 1C). The HDR-corrected fraction increased over the course of the culture, likely reflecting a selective advantage of *Prkdc*-proficient cells over indel-bearing cells. HDR frequencies were lowest (11% ± 2%) in the most stringently defined HSC population (CD201^+^CD150^+^KL) and increased in fractions with lower HSC enrichment, in line with previous reports (Figure S1B).^2^ 1 week post-editing (day 10 of culture), most cells in the expansion cultures remained c-Kit^+^ and Lin^−^, with a majority also expressing CD150 (Figure 1D). Although the initial starting population of CD201^+^CD150^+^KL cells represented only 14.7% of expanded HSCs, absolute quantification revealed an 8.9-fold expansion (Figure 1E).

To validate functional recovery of edited SCID HSCs, we transplanted 0.5 × 10^6^ expanded bulk HSC cultures into irradiated CB17/SCID recipients 7 days post-editing. B220^+^ B cells as well as CD4^+^ and CD8^+^ T cells could be detected in peripheral blood (PB) samples from 4 weeks post-stem cell transplantation (SCT) (Figure 1F). Spleens of transplanted mice contained high fractions of B and T cells (B220^+^: 36%, CD4^+^: 16%, CD8^+^: 6% of splenocytes, Figure S1C). We further found that thymocytes of transplanted mice were abundant with CD4^+^CD8^+^ double positive (DP), CD4^+^, and CD8^+^ single positive (SP) cells (Figure S1D) and thymus histology showed cortical and medullary regions (Figure S1E). The distributions of lymphocyte populations in the spleen and thymus were similar to those in age matched CB17/WT mice, suggesting orthotopic development of B and T lymphocytes. Secondary transplantations confirmed that LT-HSCs had been successfully edited in our gene correction model (Figure 1F).

As mentioned above, the high frequency of indel and SCID alleles in the transplanted HSPC population is a key limitation of this straightforward bulk expansion approach (Figure 1C). To check how this distribution was reflected in mature cell lineages, we sequenced PB cells 16–20 weeks post-SCT. As expected, *Prkdc*^HDR^ frequencies were high in lymphocytes (B220^+^: 69%, CD4^+^: 70%, CD8^+^: 63%), suggesting at least monoallelic correction in these populations (Figure 1G). Since noncorrected cells fail to complete lymphocyte development in this model, their high prevalence in the transplanted graft did not obstruct the rescue of these mature compartments. By contrast, myeloid cells, which are not subject to the same selective pressure, showed a high rate of on-target indels (*Prkdc*^indel^, 52%) and a low frequency of *Prkdc*^HDR^ alleles (14%, Figure 1G). Off-target analysis of bulk-expanded HSCs showed a low but substantial prevalence of indels (Figure 1H; Table S1). Recent studies suggest that Cas9 gene-editing produces large on-target deletions that are not captured by conventional amplicon sequencing.^7^ To check for these LDs, we adopted a droplet digital PCR (ddPCR)-based approach to assess the copy numbers of five regions spanning 1 kb around the cut site (Figure S1F).^16^ Indeed, we detected a significant drop of copy numbers not only directly around the cut site, but also several hundred bp away, indicative of LD events in the bulk-expanded population (Figure 1I).

While gene-edited HSCs effectively reversed the *Prkdc*^scid^ phenotype, these results indicate that most transplanted HSCs and their progeny contained unintended perturbations. The low allelic chimerism of *Prkdc*^HDR^ and high abundance of *Prkdc*^indel^ among myeloid cells demonstrate the challenge of ensuring that all hematopoietic cells are supplied by a genetically defined population of edited HSPCs. The potentially negative consequences of on-target indels has also recently been highlighted in other gene correction models.^17^ This drawback inspired us to establish a single-cell HSC expansion system that would allow selection of edited, *bona fide* HSCs at the clonal level.

### CD150^+^CD201^+^CD48^−^KSL cells contain clones with long-term expansion potential

Single-cell expansion of edited HSCs requires the identification of clones with prospective LT expansion potential within a population of precultured cells. Cell surface markers are particularly useful since they permit flow cytometric profiling and simultaneous cloning via fluorescence-activated cell sorting. However, HSC marker expression undergoes a dynamic shift over the course of *ex vivo* expansion.^18^^,^^19^

**Figure 2:**
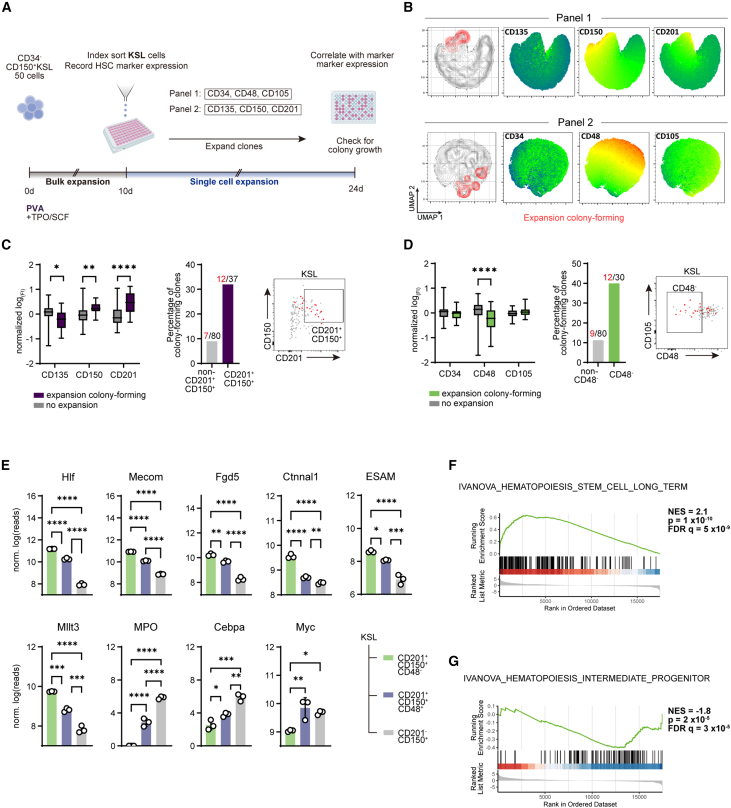
Identification of a surface marker combination for long-term (LT) expanding HSC clones (A) Experimental setup. (B) Uniform manifold approximation and projection (UMAP) representation of sorted KSL clones with overlay of panel 1 (upper) and 2 (lower) surface markers. Expansion colony-forming clones are indicated in red. (C and D) Quantification of markers associated with colony expansion. Left: fluorescence intensity (FI) measured at index sorting. Data presented as log-transformed and normalized to mean. Boxplots with whiskers showing minimum and maximum. Center: fraction of clones of the indicated phenotype showing LT expansion. Right: representative FACS sorting plots, LT-expanding clones indicated in red. (C) Panel 1 (n = 110 clones); (D) panel 2 (n = 117 clones). Multiple Mann-Whitney tests with FDR correction. (E) RNA-seq expression profiles of select HSC- and progenitor-associated genes. Error bars represent SD. One-way ANOVA with Tukey’s post-test. (F and G) Gene set enrichment analysis (GSEA) of differentially expressed genes in CD201^+^CD150^+^CD48^−^KSL (F) and CD201^−^CD150^+^KSL (G) cells. ^∗^p < 0.05, ^∗∗^p < 0.01, ^∗∗∗^p < 0.001, and ^∗∗∗∗^p < 0.0001.

To address this issue, we leveraged index sorting analysis to identify HSC markers that predict LT expansion of HSC clones. Fresh CD34^−^CD150^+^c-Kit^+^Sca-1^+^Lin^−^(CD34^−^CD150^+^KSL) HSCs were cultured for 10 days, after which KSL cells were subjected to index sorting. We used C57BL/6-derived HSCs for these experiments, since this was the background of mice used to optimize our HSC culture system and is widely used in the field.^13^ Marker profiles of each sorted KSL cell were compared with HSC colony formation after 14 days. Expression of a total of six HSC markers within the KSL population, divided into two panels (CD34, CD48, and CD105; as well as CD135, CD150, and CD201), were evaluated (Figure 2A). Colony formation was observed in 17.1% of sorted KSL clones (set 1: 16.2%, set 2: 19.1%), mainly from clones within the CD48^−^, CD150^+^, and CD201^+^ KSL populations (Figure 2B). Quantification of expression levels confirmed significantly higher expression of CD150 and CD201 as well as lower expression of CD48 among colony-forming HSCs (Figures 2C and 2D). CD135 expression was lower in colony-forming HSCs (Figure 2C); however, the small absolute difference in expression precludes the use of this marker for effective gating.

We next performed RNA sequencing to characterize the populations defined by these markers in 10-day bulk-expanded HSPCs (Figure S2A). Comparing global expression profiles, we found the greatest difference between CD201^+^CD150^+^CD48^−^ and CD201^−^CD150^+^KSL cells, with CD201^+^CD150^+^CD48^+^KSL cells representing an intermediary phenotype (Figures S2B and S2C). This representation was mirrored in the expression profiles of canonical genes related to hematopoiesis: transcripts of HSC-associated genes, such as *Hlf*, *Mecom*, and *Fgd5*, were more abundant in CD201^+^CD150^+^CD48^−^KSL cells, whereas downstream progenitor-associated genes (*MPO*, *Cebpa*) were upregulated in CD201^−^CD150^+^KSL cells (Figure 2E). Enrichment analysis confirmed that the transcriptional phenotype of CD201^+^CD150^+^CD48^−^KSL cells was similar to that of LT-HSCs (Figure 2F), while CD201^−^CD150^+^KSL cells were similar to progenitor cells (Figure 2G). GO term enrichment pointed to a proliferating state of CD201^−^CD150^+^KSL cells, with several enriched mitosis- and translation-related pathways (Figure S2D), matching our previous observation that progenitor cells proliferate more rapidly than primitive HSCs in culture (Figure 1E).

These results indicate that CD150^+^CD201^+^CD48^−^KSL cells possess LT expansion potential and retain a transcriptional phenotype associated with *bona fide* HSCs after extended culture. We thus considered these cells suitable for single-cell cloning and expansion. However, we found that the CD201^+^CD150^+^CD48^−^ expression profile was lost in single-clone-derived colonies generated from this population after 14 days, suggesting that repopulating activity had been compromised (Figure S2E). Since we have shown previously that HSC marker expression is preserved in bulk cultures even after 28 days of expansion,^13^ we reasoned that the single-cell cloning step and expansion conditions, rather than the total length of *ex vivo* expansion, were not supported by our expansion system.

### Soluplus is a superior alternative to PVA for single-cell HSC expansion

Having established that HSC activity among bulk cultured cells is enriched in the CD150^+^CD201^+^CD48^−^KSL population but that PVA-based culture conditions poorly supported their clonal expansion after re-sorting, we sought to improve clonal expansion culture conditions by screening alternative serum replacement compounds. We cultured 50 freshly isolated CD34^−^KSL cells in media supplemented with recombinant albumin, PVA and 7 different polymers and evaluated cell growth after 1 week. Of all compounds tested, only Soluplus led to comparable levels of proliferation as PVA and recombinant albumin (Figure S3A). Soluplus is an amphiphilic polyvinyl caprolactam-acetate polyethylene glycol (PCL-PVAc-PEG) graft copolymer approved for clinical use as a drug solubilizer.^20^ We hypothesized that Soluplus, like PVA,^21^ enhances the stability of cytokines in the culture medium. Indeed, we found that thrombopoietin (TPO) levels were higher in 3-day cultures if Soluplus is present compared with plain or PVA-supplemented medium (Figure S3B). To identify the most suitable concentration for HSC culture, we performed transplantations with HSCs grown in titrated concentrations of Soluplus. Although 16-week chimerism was highest in the 0.2% cohort, (Figure S3C), we selected 0.1% Soluplus for our expansion system since supplementation with 0.2% Soluplus led to mild precipitation during culture, obscuring the visibility of cells.

**Figure 3:**
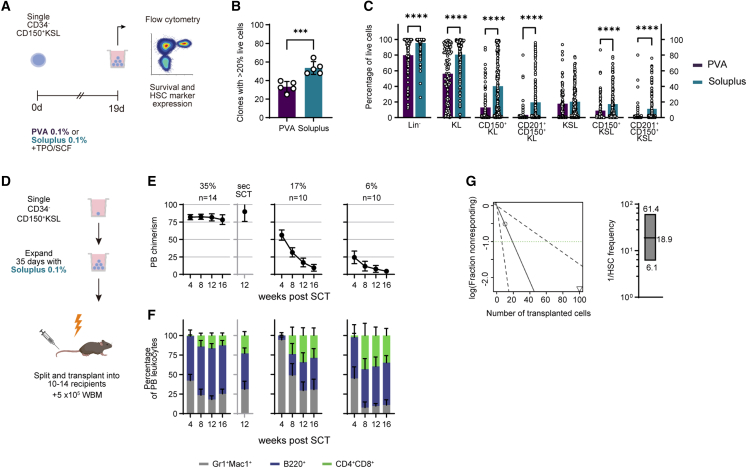
Optimization of polymer-based cultures for single-cell HSC expansion (A) Scheme of experimental setup. (B) Percentage of colonies with ≥20% live cells (n = 5 experiments). Unpaired, two-tailed t test. (C) Percentage of phenotypic HSC populations in live colonies cultured in PVA (n = 94)- and Soluplus (n = 155)-based media. Multiple Mann-Whitney tests with FDR correction. (D) Schematic of split-clone transplantation. (E and F) Donor PB chimerism (E) and lineage distribution (F) in 3 recipient groups transplanted with split clones. Numbers over graphs in (E) represent percentage of CD201^+^CD150^+^KSL cells in the transplanted clone (%) and the number of recipients (n). Secondary SCT was performed with the group showing highest chimerism, data shown in graph with gray axis. (G) Left: ELDA output of HSC frequency calculation. Right: boxplot represents calculated reciprocal mean, upper, and lower limits of HSC frequency. See also Figure S3 and Table S2. Error bars represent SD. ^∗∗∗^p < 0.001, ^∗∗∗∗^p < 0.0001.

To validate the cell growth supporting properties of Soluplus, we directly compared single HSC expansion conditions using Soluplus and PVA. Freshly isolated, single CD34^−^CD150^+^KSL cells from C57BL/6 mice were cultured in individual wells on 96-well plates. After 19 days, expansion was evaluated by flow cytometric profiling including cell viability and HSC marker expression (Figure 3A). Cell viability was higher in clones cultured in Soluplus-supplemented medium, as measured by propidium iodide (PI) exclusion staining (Figure S3D). Accordingly, the percentage of cloned HSCs forming viable cell colonies (i.e., >20% live cells) was higher under Soluplus expansion conditions (Figure 3B). Furthermore, we found that Soluplus supplementation was associated with a higher retention of HSC marker expression. In particular, the fraction of CD201^+^CD150^+^KSL cells was higher in clones cultured in Soluplus-containing medium, suggesting that Soluplus was superior in expanding phenotypically primitive HSCs (Figures 3C and S3E).

We next asked if HSC clones cultured in Soluplus medium produce functional HSC grafts *in vivo*. Freshly isolated CD45.1^+^CD34^−^CD150^+^KSL HSCs were cloned and cultured for 35 days. Three clones containing 35%, 17%, and 6% CD201^+^CD150^+^KSL cells were selected for split-clone transplantation into 10 to 15 CD45.2^+^ recipients against 5 × 10^5^ WBM cells (Figure 3D). All recipients showed multilineage LT engraftment at ≥1% chimerism in PB samples despite the high number of recipients per clone (Figures 3E and 3F), though chimerism declined significantly over time in two of the three groups. This decline was pronounced in those mice receiving grafts with a smaller CD201^+^CD150^+^KSL fraction. Secondary transplantations from pooled bone marrow of highly chimeric mice showed stable engraftment of CD45.1^+^ cells in all recipients (Figures 3E and 3F).

To quantify the potential for single HSC expansion with Soluplus, we performed a limiting dilution assay (LDA) with a CD34^−^CD150^+^KSL clone expanded for 28 days (6.37 × 10^5^ cells) and containing 84% of CD201^+^CD150^+^KSL cells (Figure S3F). We observed multilineage chimerism of ≥1% in all dose groups including from just 10 cells (in 2/5 recipients, Figures S3G and S3H). Based on these results, we estimated a mean HSC frequency of 1/18.9 (confidence interval [CI] 1/6.1–1/61.4) in the culture using extreme limited dilation analysis (ELDA, Figure 3G)^22^ and determined that the initial HSC had expanded >33,000-fold (range 10,375- to 104,426-fold corresponding to frequency CIs) under our culture conditions. Thus, our results suggest that Soluplus is superior to PVA in supporting efficient expansion of single HSCs. Importantly, whole-exome sequencing (WES) of a single-clone-derived HSC colony expanded for 28 days did not reveal nonsynonymous mutations in critical genes (Table S2). Based on these encouraging results, we attempted to expand precultured and gene-edited HSC clones.

### Soluplus enables single-cell expansion of edited HSCs

**Figure 4:**
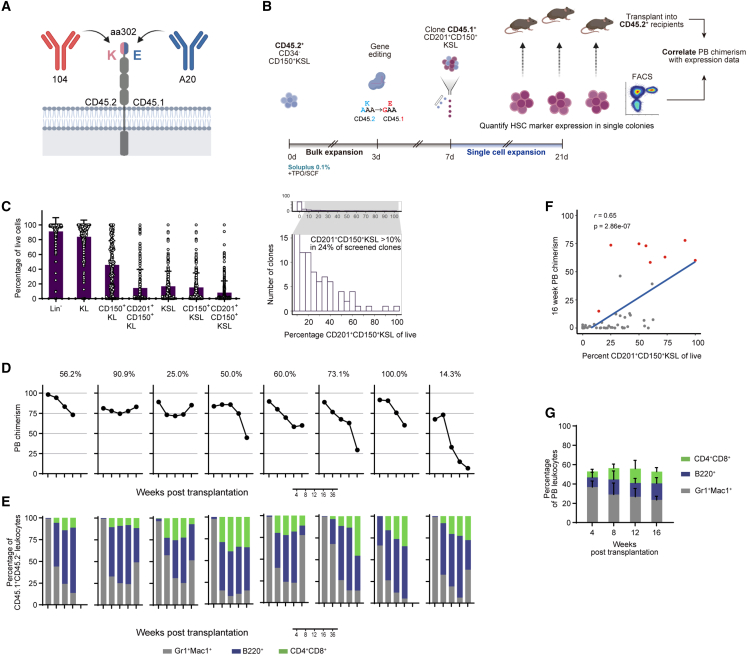
Single-cell cloning of gene-edited functional HSCs (A) Schematic showing the extracellular domain of CD45 with allele-specific antibody clones 104 and A20 and the epitope-defining amino acid. (B) Experimental setup of the single-cell editing and expansion experiment. (C) Left: fractions of CD201^+^CD150^+^KSL cells in single-cell-derived cultures 14 days after cloning (n = 261 clones). Right: Histogram of CD201^+^CD150^+^KSL cell frequency. Zoomed-in region shows clones with >10% CD201^+^CD150^+^KSL cells. (D and E) CD45.1^+^ donor PB chimerism (D) and lineage distribution (E**)** in single recipients with long-term (LT) engraftment ≥5% and multilineage reconstitution (n = 8). Numbers over graphs in (D) represent percentage of CD201^+^CD150^+^KSL cells in the transplanted clone (%). (F) Linear correlation plots of CD201^+^CD150^+^KSL cell frequency and 16-week donor chimerism. Red dots indicate LT repopulating and multilineage clones. Pearson correlation. (G) CD45.1^+^ PB chimerism and lineage distribution in secondary recipients (n = 5). See also Figure S4 and Table S3. Error bars represent SD.

To evaluate HSC gene editing and clonal expansion with single allele resolution, we developed a strategy that targets protein tyrosine phosphatase receptor type C (*Ptprc*), a cell surface protein. Two alleles of the *Ptprc* gene are common among major inbred mouse strains: *Ptprc*^a^ and *Ptprc*^b^, which code for CD45.1 (Ly5.1) and CD45.2 (Ly5.2), respectively. The CD45.1 allele is expressed in SJL/J and STS/A strains, while C57BL/6 and BALB/c strains share the CD45.2 allele.^23^ Sequence diversion between these two alleles amounts to 12 base differences resulting in 5 amino acid substitutions.^24^ The epitope of CD45.1- and CD45.2-binding antibody clones A20 and 104 is defined by a single-base difference at codon 302 (based on reference transcript GenBank:NM_001111316.2, Figure 4A).^25^ We leveraged the low complexity of this single-nucleotide polymorphism (SNP) to simultaneously identify and clone gene-edited HSCs, followed by single-cell expansion and transplantation (Figure 4B). To this end, we knocked in the CD45.1-specific SNP variant (A → G, p.K302E) into the *Ptprc* gene of CD45.2^+^ HSCs (Figure S4A).

CD34^−^CD150^+^KSL HSCs from CD45.2^+^ C57BL/6 mice were cultured in Soluplus-HSC expansion medium for 3 days, after which we targeted *Ptprc* for allele conversion. 4 days after editing, 20% (±5.8%) of cells had converted to the CD45.1^+^CD45.2^−^ phenotype (Figure S4B). As with the SCID model, conversion rates were lower in the primitive CD201^+^CD150^+^KSL fraction (Figure S4C). We started 570 single-cell cultures from the CD45.1^+^CD201^+^CD150^+^KSL population. 14 days later, cell proliferation could be observed in, and appropriate flow cytometric data could be obtained from, 46% of all sorted clones (261/570). Surface marker expression was heterogeneous, with 24% (63/261) containing at least 10% of CD201^+^CD150^+^KSL cells (Figure 4C). Fifty-one colonies were selected for transplantation into single CD45.2^+^ recipients. Donor chimerism of ≥5% was observed in 29/51 (57%) and 17/51 (33%) recipients 4 and 16 weeks after transplantation, respectively (Figures 4D and S4D). Among the recipients showing LT chimerism, multilineage reconstitution (myeloid, B cell, and T cell lineages ≥5% of donor hematopoiesis) was observed in 8 recipients (16% of recipients, Figure 4E), while the remaining mice showed biased donor hematopoiesis (Figure S4E). Although myeloid contribution seemed relatively low in some LT-engrafted recipients (Figure 4E), a comparison with the co-transplanted competitor graft revealed similar lineage contributions, suggesting an assay-specific phenomenon rather than a linage bias of the expanded HSC clones (Figure S4F). Linear correlation analysis of pre-SCT marker expression and 16-week chimerism revealed several parameters associated with LT engraftment, the strongest of which was the fraction of CD201^+^CD150^+^KSL cells in the transplanted graft (Figures 4F and S4G). Secondary transplantations were performed with whole bone marrow cells from a highly chimeric primary recipient. Analysis of bone marrow cells revealed high chimerism of 77% within the KSL population (Figure S4H). 16 weeks after secondary transplantation, multilineage PB donor chimerism was observed in all secondary recipients (Figure 4G). WES analysis of gene-edited CD45.1^+^CD45.2^−^ cells obtained from the BM of these secondary recipients did not indicate nonsynonymous variants in oncogenic driver genes (Table S3).

Together, these results established that HSCs can be gene edited and clonally expanded while maintaining their self-renewal properties using our expansion system. Our experiments also confirm the expression of CD201 and CD150 on expanded clones as predictive of LT engraftment. This approach therefore provided the framework for probing single HSC clones for on- and off-target edits prior to transplantation.

### Single-cell expansion of edited HSCs permits the assembly of a genetically defined HSC graft

**Figure 5:**
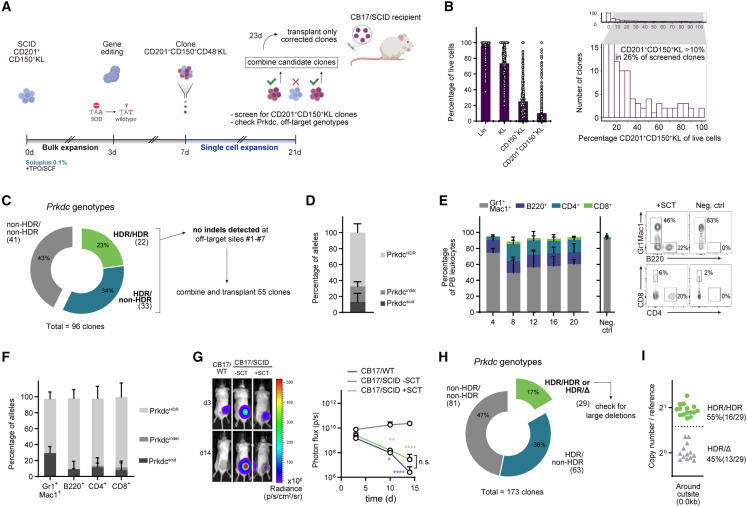
Autologous HSCT using gene-corrected HSC clones is curative in an immunodeficiency mouse model (A) Schematic of the single clone *Prkdc*^scid^ correction model. (B) Single-cell SCID HSC expansion outcomes. Left: frequencies of phenotypic HSC populations in screened colonies (n = 384 from 3 experiments). Right: histogram of CD201^+^CD150^+^KL cell frequency. Enlarged region shows clones with ≥10% CD201^+^CD150^+^KL cells. (C) Genotyping of candidate clones (Sanger sequencing) (n = 96 clones, 3 experiments). Only clones with at least one HDR-corrected allele were sequenced at the off-target loci. (D) Allelic composition of the combined cell mixture at the edited *Prkdc* locus (n = 3). (E) Frequencies of PB leukocytes in CB17/SCID recipients. Left: lineage distributions in treated mice (n = 3) and in recipients receiving only 2 × 10^5^ CB17/SCID whole bone marrow cells (neg. ctrl., n = 3). Right: representative FACS plots at 16 weeks post-SCT. (F) Allele frequencies in sorted PB cells 20 weeks post-SCT (n = 3 mice from 3 experiments). (G) Xenograft transplantation assay. A549 cells expressing the luminescent reporter Akaluc were injected subcutaneously (s.c.) and tumor growth was tracked by *in vivo* imaging. Left: representative images from CB17/WT, transplanted CB17/SCID, and untreated CB17/SCID mice 3 and 14 days after inoculation. Right: quantification of luminescence over a 14-day period (CB17/WT: n = 4, CB17/SCID − SCT: n = 3, CB17/SCID + SCT: n = 3). Two-way ANOVA and Tukey’s multiple comparison test. (H) Genotyping of candidate clones (Sanger sequencing) (n = 173 clones, 2 experiments). Clones producing a single, HDR-corrected sequencing trace were checked for LD (n = 29). *Prkdc*^HDR/HDR^, homozygous correction; *Prkdc*^HDR/Δ^, hemizygosity. (I) *Prkdc* copy-number analysis using 0.0 kb *Prkdc* (around cutsite) probe, quantified against reference gene. See also Figure S5. Error bars represent SD. ^∗^p < 0.05, ^∗∗^p < 0.01, and ^∗∗∗∗^p < 0.0001.

To explore this approach, we adopted our single-cell expansion platform to the SCID immunodeficiency model. Analogous to our previous experiments, we expanded SCID HSCs in Soluplus-supplemented expansion medium and edited SCID HSCs to correct the *Prkdc*^scid^ mutation. After editing, CD201^+^CD150^+^CD48^−^KL cells were cloned by flow cytometry and expanded for 14 days (Figure 5A). Since expression of CD201 and CD150 was predictive of LT engraftment, we first screened for colonies containing a CD201^+^CD150^+^KL population of over 10% and then checked for the presence of the corrected allele (*Prkdc*^HDR^) and absence of off-target mutations. Candidate clones were then combined and administered to a SCID recipient (Figure 5A).

Phenotypic profiling data could be obtained from 19% (384) of sorted clones (Figure 5B). Of these, 26% (99/384) contained a population of CD201^+^CD150^+^KL HSCs ≥10%, which we classified as transplantable clones. Correlating HSC marker expression on the founder cell with the outcome of the expansion cultures, we observed that CD201^hi^/CD150^hi^ cells were more likely to, and that CD105^−^ cells did not, generate transplantable clones (Figure S5A). Sequencing of all intended loci (*Prkdc*, off-targets #1–7) could be achieved in most of these clones (96/384). We detected *Prkdc*^HDR^ in 57% (55/96) of genotyped clones, and all corrected clones were free of off-target mutations at predicted sites (Figures 5C and S5B). As a result, an average of 18 HDR^+^Off-target^−^ colonies were selected for transplant per experiment. Due to this selection step, the combined allelic composition of the selected clones was dominated by *Prkdc*^HDR^ alleles (67%, Figure 5D). This stands in contrast to our bulk-transplant approach, in which indel alleles were most abundant (Figure 1C). In PB samples from transplanted SCB17/SCID mice, we detected B and T lymphocytes from week 4 through week 20 post-transplant, confirming LT engraftment (Figures 5E and S5E). In contrast to our bulk-transplant experiments, the *Prkdc*^HDR^ allele was highly prevalent not only in lymphoid, but also in myeloid cells (>60%, Figure 5F). Notably, *Prkdc*^indel^ frequency was low in all PB lineages.

Having achieved robust reconstitution of lymphoid cells, we asked if correction of the *Prkdc*^scid^ allele also led to development of a functional immune system. Double (CD4^+^CD8^+^) and single (CD4^+^ and CD8^+^) -positive cells were detected among thymocytes of SCID recipients (Figure S5C). Length diversity of the third complementary determining region (CDR3) in the T cell receptor gene is a direct function of *Prkdc* activity. We measured CDR3 region length distributions within multiple T cell receptor beta chain (*Tcrb*) gene families of splenic CD4^+^ T cells using spectratype analysis.^26^ Distribution profiles showed Gaussian distribution patterns without oligoclonal spikes (Figure S5D), suggesting that restored *Prkdc* activity permitted the generation of unbiased CDR3 regions. To confirm the status of immune cell function *in vivo*, we immunized transplanted CB17/SCID mice with a T-dependent antigen, nitroiodophenyl (NIP)-conjugated OVA (NIPOVA), intraperitoneally (i.p.).^27^ NIP_30_-specific IgG and IgM levels were significantly elevated in the serum of transplanted CB17/SCID mice 19 days post-immunization (p.i.), indicative of a specific humoral immune response (Figure S5F). On the other hand, untreated CB17/SCID mice showed no specific response. To assess cellular immunity, we inoculated mice with the human lung cancer cell line A549, hypothesizing that reconstitution of immunity would trigger xenograft rejection. Indeed, rejection was observed in transplanted mice only, whereas progressive tumor growth was detected in non-transplanted SCID mice (Figure 5G). We therefore conclude that the molecular and functional hallmarks defining the *Prkdc*^scid^ phenotype had been reversed. These results illustrate that a functional graft can be assembled from individually expanded and profiled gene-edited HSC clones.

As demonstrated above (Figure 1I), *Prkdc* editing frequently results in the production of LDs around the cut site. At the single-clone level, these events lead to loss of heterogeneity (LOH) and may remain undetected upon genotyping if one of the priming sites is involved. Consequently, some HSC clones will appear to contain two HDR-corrected alleles (homozygous correction, *Prkdc*^HDR/HDR^) but will actually harbor only one (*Prkdc*^HDR/Δ^, Figure S5G). To confirm that our system allows for the detection of these clones, we sequence-genotyped 173 Prkdc-edited colonies containing ≥10% CD201^+^CD150^+^KL HSCs. Of these, 29 (17%), produced a single, HDR-corrected sequencing trace (Figure 5H). Subsequent quantification of alleles using the Prkdc-0.0 kb probe revealed that 16 of the clones harbored two copies (HDR/HDR), whereas 13 clones were indeed hemizygous (HDR/Δ), indicative of LDs (Figures 5I and S5H). Thus, our approach enables the detection and exclusion of clones containing LDs in gene-edited HSC colonies.

### Controlling genetic heterogeneity in gene-edited human HSCs

**Figure 6:**
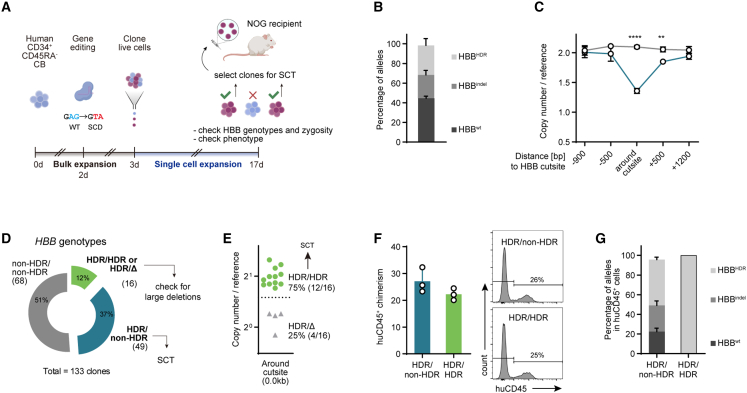
Controlling genetic heterogeneity in gene-edited human HSCs (A) Scheme of experimental setup. (B) Post-editing allele distribution at the *HBB* locus, assessed by ICE (n = 3 cultures). (C) Copy-number analysis of *HBB* probes against reference gene (n = 3). Two-way ANOVA with Sidak’s multiple comparison test. (D) Genotyping of candidate clones (Sanger sequencing) (n = 133 clones, 2 experiments). Heterozygous clones were combined for SCT. Clones producing a single, HDR-edited sequencing trace were checked for LD (n = 16). (E) Copy-number analysis of *HBB* probe 0.0 kb against reference gene. (F) Left: spleen huCD45^+^ chimerism in *HBB*^HDR/non-HDR^ and *HBB*^HDR/HDR^ groups (n = 3 each). Right: representative FACS plots. (G) Percentage of HDR alleles in huCD45^+^ cells sorted from bone marrow and spleens of *HBB*^HDR/non-HDR^ and *HBB*^HDR/HDR^ engrafted mice (n = 3 each). ^∗∗^p < 0.01, ^∗∗∗∗^p < 0.0001.

To demonstrate the potential of our clonal expansion concept in the human setting, we edited healthy cord blood (CB)-derived CD34^+^CD45RA^−^ human HSCs (huHSCs) at the *HBB* locus to knock in a two-base mutation, creating a sickle cell disease (SCD)-like genotype (Figure S6A). Analogous to our murine protocol, single cells were cloned and cultured for 2 weeks followed by genetic profiling, selection, and xenotransplantation (Figure 6A). Since *bona fide* HSCs cannot be expanded from single huHSCs using currently available culture protocols, we adopted a single-cell differentiation and proliferation assay to produce clonally derived HSPC colonies.^28^ At the time of cloning, around one third of alleles (30% ± 7%) contained the HDR edit (*HBB*^HDR^, Figure 6B). Though not to the extent seen in murine *Prkdc*^SCID^ HSCs, we did detect copy-number losses at the HBB locus in edited bulk-expanded huHSCs, suggestive of LDs (Figures 6C and S6B). The SCD edit was detected in about half of the sequence-genotyped clones (49%, 65/133) (Figure 6D). Phenotypically, these colonies were dominated by erythro-myeloid lineages (Figures S6C ans S6D). We separated these clones into 2 groups: those heterozygous for the SCD edit (*HBB*^HDR/non-HDR^) were selected for immediate xenotransplantation, while clones that produced a single, HDR-corrected sequencing trace (*HBB*^HDR/HDR^ or *HBB*^HDR/Δ^) were subjected to copy-number profiling. We found that while the majority of these clones were homozygous, four out of 16 in fact only had one allele, indicating a *HBB*^HDR/Δ^ genotype (Figure 6E). The *HBB*^HDR/HDR^ colonies were selected for xenotransplantation. Human donor (huCD45^+^) splenic chimerism 7–10 days after transplantation was similar at 20%–30% in both groups (Figure 6F). We sorted huCD45^+^ cells from recipient spleens and bone marrows and sequenced them at the HBB locus. As expected, cells in the heterozygous (*HBB*^HDR/non-HDR^) and homozygous (*HBB*^HDR/HDR^) transplant groups carried around 50% and 100% HDR alleles, respectively (Figures 6G and S6E). Therefore, our method has allowed us to select and transplant human graft cells with an entirely HDR-corrected genotype.

## Discussion

The platform established here permits clonal expansion, direct selection, and transplantation of a primary adult tissue stem cell population without the use of pluripotent intermediary cells. We believe our results not only have important implications for studying the genetics of hematopoiesis but also highlight the potential of this approach for gene therapy. With the widespread interest in gene editing as a therapeutic modality, concerns about hazardous mutations in edited cell products have become more visible.^29^ Our expansion system addresses this concern in the murine model by enabling marker-free selection of edited LT-HSC clones with known mutational profiles. As shown here, this not only includes on- and off-target indels, but also long deletions, a recently identified by-product of Cas9 gene editing.^7^ Importantly, our approach is not restricted to Cas9-induced aberrations but can be used to exclude from transplantation clones bearing any kind of collateral genetic lesion (e.g., adeno-associated virus [AAV]-associated insertions^30^).

The addition of Soluplus facilitated the expansion of HSC colonies to previously unattainable levels of over 30,000-fold. *In vivo*, this translated to high levels of chimerism in split-clone transplantations. The mechanism by which Soluplus, a biocompatible excipient for oral drug formulations, exerts its HSC-supportive properties remain largely undefined, although we did find that it enhances the stability of TPO during culture. This phenomenon likely extends to other cytokines and essential factors in the medium as well.^20^^,^^31^ Intriguingly, we have recently found that Soluplus supplementation also improved *in vitro* proliferation of human CB-derived CD34^+^ cells compared with PVA (55- versus 10-fold over 30 days) in a cytokine-free, small-molecule-based expansion system.^32^

Previous studies have achieved molecular reversion of the *Prkdc*^scid^ mutation in Lin^−^ bone marrow cells, but low efficiencies have obstructed effective functional correction of immunodeficiency *in vivo*.^33^^,^^34^ This might be attributed to the higher occurrence of mutagenic repair events, such as microhomology-mediated end joining (MMEJ). Our demonstration that a sufficient corrected cell dose can be generated despite this challenging background highlights the utility of HSC expansion to propagate corrected cells *ex vivo*. Such an approach would also be highly relevant to settings where on-target mutations are detrimental and limit the curative potential of gene correction approaches, e.g., correction of the hemoglobin sickle allele.^17^ To demonstrate this, we apply our expansion-selection strategy to a human *HBB* editing model and illustrate the feasibility to generate fully HDR-corrected cell grafts. In summary, we have developed an easily adoptable and powerful clonal expansion platform for precise genetic and functional interrogation of murine HSCs at the single-cell level.

Although individual fold expansion rates were very high using our culture conditions, the overall yield of transplantable HSC clones was low, with only ∼3% of sorted clones selected for transplantation. The fact that many HSCs did not produce colonies at all highlights the importance of identifying *bona fide* HSCs among precultured cells. CD201 is a useful marker indicating repopulation potential.^35^ Indeed, we found that the CD201^+^CD150^+^CD48^−^KSL phenotype was most likely to contain LT-expanding clones, but additional markers are needed to further resolve LT-HSC activity in expansion cultures. Furthermore, in those HSC clones that do expand over 14 days, the majority still lose the CD201^+^CD150^+^KSL phenotype even with our optimized protocol, pointing to a need to further improve expansion conditions. Clonal yield might also be affected by the gene editing strategy. In our experiments, we used single-strand oligonucleotides (ssODNs) to deliver HDR templates, which preserve the clonogenicity of HSCs compared with viral vectors (AAV and integration-deficient lentiviral vector (IDLV)) by inducing a lower p53 response.^30^^,^^36^ It is likely that using these more genotoxic delivery methods would further impact the clonal yield due to increased cellular stress responses. On the other hand, it would be interesting to investigate if non-DSB or template-free editing tools, such as base or prime editors, enhance colony-forming potential.^37^^,^^38^ Likewise, the use long ssODNs of up to 2,000 nucleotides might enable the delivery of long templates without eliciting the stress response associated with viral vectors, although this has not been experimentally verified.

An important limitation of single-cell-expanded HSCs for SCT is the oligoclonal composition of the transplanted graft, which is a direct consequence of the low clonal yield frequencies discussed above and raises concerns regarding clonal dominance. While these concerns are warranted, it is important to consider the impact of Cas9 gene editing itself on the clonal composition of HSC pools *in vivo*. A recent study by Ferrari et al. has shown that the LT repopulating graft arising from a Cas9-edited huHSC pool is dominated by less than 10 clones after transplantation into NSG mice.^39^ Similarly, Sharma et al. have reported that a median of 2 clones contribute to 50% of allele diversity in an *HBB* gene editing model.^40^ Although these observations, which were obtained from xenograft models, may not accurately inform our understanding of clonal dynamics in the autologous setting, they do reflect the negative impact of Cas9 gene editing, mediated by DSB-induced p53 induction, on HSC clonal diversity. Thus, one can speculate that our screening process based on the CD201^+^CD150^+^CD48^−^KSL phenotype selects for clones that would have dominated LT hematopoiesis even if the bulk population had been transplanted.

### Limitations of the study

Regarding applicability to huHSCs, the major obstacles to applying our approach currently are the limited expansion efficiency of huHSCs and the lack of reliable markers to identify and purify LT-expanding huHSC clones. Regarding the former, current culture protocols unfortunately do not support single-cell expansion of huHSCs. For this reason, we used a differentiation and proliferation protocol to demonstrate the advantages of our expansion-selection platform in the human system. Furthermore, our work shows that sorting populations highly enriched for HSC activity (CD201^+^CD150^+^CD48^−^KSL) is critical in post-gene editing cloning and expansion. There is a pressing need to identify reliable markers of LT-HSC activity especially in the human system, where HSC frequencies among precultured cells is ∼1:38 among the most stringently defined population (CD34^+^CD45RA^−^CD90^+^CD133^+^EPCR^+^ITGA3^+^).^41^ The diverse genetic background in humans raises additional challenges to identifying stable markers and developing reliable enrichment strategies across individuals. To this end, techniques that link clonal phenotype with expansion outcome, like index sorting and single clone transplantation, will be essential.^35^ Moreover, we recognize that our investigations concerning the function of Soluplus are mainly observational and acknowledge the need for further mechanistic studies.

With a view toward a hypothetical clinical application of our approach, the above issues of low clonal yield and graft oligoclonality are compounded by the high number of clones required for polyclonal reconstitution in humans and represent a major obstacle to translation. In our opinion, improving human expansion efficiencies to levels similar to the murine system, together with the identification of reliable HSC markers, will be key to apply our concept to the human system.

## STAR★Methods

### Key resources table

**Table:** 

REAGENT or RESOURCE	SOURCE	IDENTIFIER
**Antibodies**		

anti-mouse Ckit-APC (2B8)	Thermo Fisher Scientific	Cat#17-1171-83; RRID:AB_469431
anti-mouse CD4-APC (RM4-5)	Thermo Fisher Scientific	Cat#17-0042-83; RRID:AB_469324
anti-mouse CD8a-APC (53-6.7)	Thermo Fisher Scientific	Cat#17-0081-83; RRID:AB_469336
anti-mouse CD48-APC (HM48-1)	Thermo Fisher Scientific	Cat#17-0481-82; RRID:AB_469408
anti-mouse CD135-APC (A2F10)	Thermo Fisher Scientific	Cat#17-1351-82; RRID:AB_10717261
anti-mouse CD45.1-APC/Cy7 (A20)	Tonbo Biosciences	Cat#25-0453; RRID:AB_2621629
anti-mouse Sca-1-APC/Cy7 (D7)	BioLegend	Cat#108126; RRID:AB_10645327
anti-mouse CD45R(B220)-APC/eFluor780 (RA3-6B2)	Thermo Fisher Scientific	Cat#47-0452-82; RRID:AB_1518810
anti-mouse Ckit-APC/H7 (2B8)	BD Biosciences	Cat#560185; RRID:AB_1645231
anti-mouse CD4-Biotin (RM4-5)	Thermo Fisher Scientific	Cat#13-0042-85; RRID:AB_466330
anti-mouse CD8a-Biotin (53-6.7)	Thermo Fisher Scientific	Cat#13-0081-85; RRID:AB_466347
anti-mouse CD45R(B220)-Biotin (RA3-6B2)	Thermo Fisher Scientific	Cat#13-0452-82; RRID:AB_466449
anti-mouse TER119-Biotin (TER119)	Thermo Fisher Scientific	Cat#13-5921-85; RRID:AB_466798
anti-mouse Gr1-Biotin (RB6-8C5)	Thermo Fisher Scientific	Cat#13-5931-85-85; RRID:AB_466801
anti-mouse CD11b-Biotin (M1/70)	Thermo Fisher Scientific	Cat#13-0112-85; RRID:AB_466360
anti-mouse CD127-Biotin (A7R34)	Thermo Fisher Scientific	Cat#13-1271-85; RRID:AB_466589
anti-mouse CD45.2-BV421 (104)	Thermo Fisher Scientific	Cat#48-0454-82; RRID:AB_11042125
anti-mouse CD34-FITC (RAM34)	Thermo Fisher Scientific	Cat#11-0341-85; RRID:AB_465022
anti-mouse CD45.2-FITC (104)	BioLegend	Cat#109806; RRID:AB_313443
anti-mouse CD48-FITC (HM48-1)	BioLegend	Cat#103404; RRID:AB_313019
anti-mouse CD4-FITC (RM4-5)	Tonbo Biosciences	Cat#35-0042; RRID:AB_2621666
anti-mouse CD8a-FITC (53-6.7)	Thermo Fisher Scientific	Cat#11-0081-85; RRID:AB_464916
anti-mouse CD45R(B220)-FITC (RA3-6B2)	Thermo Fisher Scientific	Cat#11-0452-85; RRID:AB_465055
anti-mouse TER119-FITC (TER119)	Thermo Fisher Scientific	Cat#11-5921-82; RRID:AB_465311
anti-mouse Gr1-FITC (RB6-8C5)	Thermo Fisher Scientific	Cat#11-5931-82; RRID:AB_465314
anti-mouse CD11b-FITC (M1/70)	Thermo Fisher Scientific	Cat# 11-0112-82; RRID:AB_464935
anti-mouse CD4-PB (RM4-5)	BioLegend	Cat#100531; RRID:AB_493374
anti-mouse CD8a-PB (53-6.7)	BD Biosciences	Cat#558106; RRID:AB_397029
anti-mouse CD45R(B220)-PB (RA3-6B2)	BD Biosciences	Cat#558108; RRID:AB_397031
anti-mouse TER119-PB (TER119)	BioLegend	Cat#116231; RRID:AB_2149212
anti-mouse Gr1-PB (RB6-8C5)	BioLegend	Cat#108430; RRID:AB_893556
anti-mouse CD11b-PB (M1/70)	BioLegend	Cat#101224; RRID:AB_755986
anti-mouse Sca-1-PE (D7)	BD Biosciences	Cat#12-5981-83; RRID:AB_466087
anti-mouse CD201-PE (eBio1560)	Thermo Fisher Scientific	Cat#12-2012-82; RRID:AB_914317
anti-mouse Gr1-PE (RB6-8C5)	Thermo Fisher Scientific	Cat#12-5931-83; RRID:AB_466046
anti-mouse CD11b-PE (M1/70)	BD Biosciences	Cat#557397; RRID:AB_396680
anti-mouse CD105-PE (MJ7/18)	Thermo Fisher Scientific	Cat#12-1051-82; RRID:AB_657524
anti-mouse CD150-PE/Cy7 (TC15-12F12.2)	BioLegend	Cat#115913; RRID:AB_439796
anti-mouse CD45.1-PE/Cy7 (A20)	Tombo Biosciences	Cat#60-0453; RRID:AB_2621850
anti-mouse CD8a-PE/Cy7 (53-6.7)	BioLegend	Cat#100722; RRID:AB_312761
anti-mouse Sca-1-BV605 (D7)	BioLegend	Cat#108133; RRID:AB_2562275
Streptavidin-APC/eFluor780	Thermo Fisher Scientific	Cat#47-4317-82; RRID:AB_10366688
Streptavidin-BV421	BioLegend	Cat#405225
anti-human CD41-BV421 (HIP8)	BioLegend	Cat#303730; RRID:AB_2629627
anti-human CD235a-FITC (HI264)	BioLegend	Cat#349104; RRID:AB_10613463
anti-human CD71-PE (CY1G4)	BioLegend	Cat#334106; RRID:AB_2201481
anti-human CD34-APC (581)	BioLegend	Cat#343510; RRID:AB_1877153
anti-human CD33-PE/Cy7 (WM53)	Thermo Fisher Scientific	Cat#25-0338-42; RRID:AB_1907380
anti-human CD45-APC/Cy7 (HI30)	BioLegend	Cat#304014; RRID:AB_314402
anti-mouse CD45.1-eFluor 450 (A20)	Thermo Fisher Scientific	Cat#48-0453-82; RRID:AB_1272189

**Biological samples**		

Human CD34^+^ cord blood HSCs	StemExpress	Cat#CB3400.5C

**Chemicals, Peptides, and Recombinant Proteins**		

Polyvinyl alcohol (PVA), 87-90% hydrolyzed	Sigma	Cat#P8136; CAS 9002-89-5
Soluplus	BASF	CAS 402932-23-4
Recombinant Murine TPO	Peprotech	Cat#315-14; P40226
Recombinant Murine SCF	Peprotech	Cat#250-03; P20826
Insulin-Transferrin-Selenium	Thermo Fisher Scientific	Cat#41400045
Nitroiodophenyl (NIP)-conjugated OVA (NIPOVA)	Biosearch Technologies	Cat#N-5041-10

**Deposited data**		

RNA-Seq data	This paper	GEO: GSE232527
Whole exome sequencing data	This paper	BioProject: PRJNA974717
Raw figure data	This paper	Mendeley Data:https://doi.org/10.17632/br3y74vjh4.1

**Experimental Models: Cell Lines**		

Human: A549 (lung adenocarcinoma)	ATCC	Cat#CCL-185; RRID:CVCL_0023
Murine: MS-5 (bone marrow stroma)	Riken BRC	Cat#RCB4680; RRID:CVCL_2128

**Experimental Models: Organisms/Strains**		

Mouse: CB17/SCID: C.B-17/Icr-^scid/scid^Jcl	Clea	RRID:IMSR_JCL:JCL:mID-0003
Mouse: CB17/WT: C.B-17/Icr-^+/+^Jcl	Clea	RRID:IMSR_JCL:JCL:mID-0004
Mouse: CD45.2^+^ C57BL/6: C57BL/6NCrSlc	SLC	RRID:MGI:5295404
Mouse: CD45.1^+^ C57BL/6: B6.SJL-Ptprc^a^ Pepc^b^/BoyJ	Sankyo Labo	RRID:IMSR_JAX:002014

**Oligonucleotides**		

	See Table S4 for list of oligonucleotides	N/A

**Software and Algorithms**		

FlowJo version 10	BD	https://www.flowjo.com; SCR_008520
IndexSort version 0.1.6 (FlowJo plugin)	Freier^42^	https://www.flowjo.com
UMAP version 3.1 (FlowJo plugin)	McInnes et al.^43^	https://arxiv.org/abs/1802.03426
Prism version 9.1	Graphpad	https://www.graphpad.com; SCR_002798
R version 4.0.0	R Foundation	https://www.r-project.org; SCR_001905
DESeq2 version 1.32.0	Love et al.^44^	SCR_015687
clusterProfiler version 4.0.2	Wu et al.^45^	SCR_016884
ComplexHeatmap version 2.8.0	Gu et al.^46^	SCR_017270
Inference of CRISPR edits (ICE)	Synthego	https://ice.synthego.com/
Tracking of Indels by Decomposition (TIDE)	Brinkman et al.^47^	http://shinyapps.datacurators.nl/tide/
QuantaSoft Analysis Pro 1.0	Bio-Rad	https://www.bio-rad.com/

### Resource availability

#### Lead contact

Further information and requests for resources and reagents should be directed to Satoshi Yamazaki (y-sato4@md.tsukuba.ac.jp).

#### Materials availability

This study did not generate new unique reagents.

### Experimental model and subject details

#### Mice

Male C.B-17/Icr-^+/+^Jcl wildtype (CB17/WT) and male C.B-17/Icr-^scid/scid^Jcl (CB17/SCID) mice were obtained from Clea Inc., Japan. C57BL/6NCrSlc (Ly 5.2, CD45.2) mice were purchased from SLC Inc., Japan. C57BL/6-Ly5.1 (Ly 5.1, CD45.1) mice were purchased from Sankyo Labo, Japan. All mice were obtained at age 8-10 weeks and housed in specific-pathogen-free (SPF) conditions at up to 5 mice per cage, with free access to standard rodent feed and kept under a 12h light/12h dark cycle. All animal protocols were approved by the Animal Care and Use Committee of the Institute of Medical Science, University of Tokyo.

#### Primary cell cultures

All cell culture operations were conducted under sterile hoods. Cells were kept in an incubator (Panasonic) at 37°C and a constant CO_2_ fraction of 5%. For gene editing experiments, HSCs were cultured in hypoxia (5% F_i_O_2_).^48^ Cell concentrations were determined on a Countess II cytometer (Thermo Fisher Scientific) after staining with Turk’s staining buffer (bone marrow cells) or trypan blue dead stain solution. Male murine HSCs were cultured in a Ham’s F12 medium (Wako) supplemented with 10 mM HEPES (Thermo Fisher Scientific), recombinant cytokines murine TPO (100 ng/ml, Peprotech) and SCF (10 ng/ml, Peprotech), as well as insulin-transferrin-selenium (ITS, Thermo Fisher Scientific, 1:100 dilution) and 1% Penicillin-Streptomycin-L-Glutamine (PSG, Wako). Recombinant human albumin (Albumin Biosciences), polyvinyl alcohol (PVA, 84% hydrolyzed, Sigma), Kollidon 12 PF, Kollidon 17 PF, Kollidon 90 F, Poloxamer 188 Bio, Poloxamer 407 Geismar, povidone and Soluplus (all BASF) were added at a concentration of 0.1% v/v (except for Soluplus titration experiments). PVA and Soluplus-supplemented media are designated PVA-HSC and Soluplus-HSC expansion medium, respectively. Polymers were added from prepared stocks of 10% w/v in ddH_2_O. Bulk murine HSC expansions were cultured on human fibronectin-coated 24-well dishes (Corning). Single cell expansions were cultured on untreated U-bottom 96-well plates (TPP, cultures starting with 1-50 cells).

Human CD34^+^ cord blood HSCs (from a male infant born at 40 weeks, StemExpress) were cultured in human HSC bulk expansion medium, consisting of IMDM (Wako) supplemented with 10 mM HEPES, Soluplus 0.01%, and recombinant human cytokines (FLT3L 100ng/ml, G-CSF 10 ng/ml, SCF 100 ng/ml, IL-6 10 ng/ml (all from Peprotech), TPO 15 ng/ml (Shenandoah)). Recombinant cytokines, ITS, PSG and polymers were freshly added to base media before each application. Human bulk HSCs were cultured on CellBIND 24-well dishes (Corning). For human single cell expansions, CellBIND flat bottom 96-well dishes were used (Corning).

#### Cell line cultures

Human male epithelial lung cancer cell line A549 (ATCC) was cultured in DMEM (Wako) supplemented with 10% FBS (Thermo Fisher) and 1% Penicillin-Streptomycin (Wako). Murine stromal MS-5 cells were cultured in MEMα (Wako) supplemented with 10% FBS and 1% Penicillin-Streptomycin. Cells were passaged after reaching 70-80% confluency. Transduction procedure with Akaluc-expressing lentivirus is described in the method details section below.

### Method details

#### Murine HSC isolation

Male 8-10 week-old mice were sacrificed by cervical dislocation after isoflurane anesthesia. Pelvic, femur and tibia bones were isolated and crushed, and the obtained cell solution was filtered through a 48 μm nylon mesh and whole bone marrow cells were counted. Positive selection of c-Kit^+^ cells was performed with anti-APC magnetic-activated cell sorting (MACS, Miltenyi Biotec) antibodies after staining cells with c-Kit-APC antibody for 30 minutes. Enriched c-Kit^+^ cells were incubated with anti-Lineage antibody cocktail (consisting of biotinylated Gr1[LY-6G/LY-6C], CD11b, CD4, CD8a, CD45R[B220], IL7-Rα, TER119) for 30 minutes. This was followed by staining with CD34-FITC, CD201-PE (for CB17 strains) or Sca-1-PE (for C57BL/6 strains), c-Kit-APC, streptavidin-APC/eFluor 780 and CD150-PE/Cy7 antibodies for 90 minutes. CD201^+^CD150^+^c-Kit^+^Lin^-^ (CD201^+^CD150^+^KL) cells and CD34^-^CD150^+^c-Kit^+^Sca-1^+^Lin^-^ (CD34^-^CD150^+^KSL) cells from CB17 and C57BL/6 bone marrows, respectively, were sorted via fluorescence-activated cell sorting (FACS) on a Aria II cell sorter (BD) using a 100 μm nozzle and appropriate filters and settings. Propidium iodide (PI) was used to exclude dead cells. For bulk expansion of HSCs before gene editing, 5000 cells were sorted into 1 ml of HSC expansion medium per well. Medium changes were not performed until gene editing (day 3 of culture). For single cell expansion of freshly isolated HSCs, single HSCs were sorted into individual wells on a 96-well U-bottom plate (TPP) prefilled with 200 μl of culture medium. Culture medium was changed on day 7 post-sort, after which complete media changes were performed every 2-3 days.

#### Murine CRISPR/Cas9 gene editing

Seventeen micrograms of recombinant S. pyogenes Cas9 (S.p. Cas9 Nuclease V3, IDT) were complexed with single guide RNA (sgRNA, synthesized at IDT) at a molar ratio of 1:2.5 (104 pmol Cas9:260 pmol sgRNA) for 10 minutes at 25°C to form ribonucleoprotein (RNP) complexes. Sequences of sgRNA targeting *Prkdc*^scid^ (Prkdc_gRNA1) and *Ptprc*^b^ (CD45.2, Ptprc_gRNA1) are listed in Table S3. Expanded HSCs were washed twice with PBS, pelleted, and resuspended in 20 μl electroporation buffer P3 (Lonza). Cells were gently added to the RNP duplex. For knockin experiments, 200 pmol of single-strand oligonucleotide (ssODN) templates (synthesized at IDT, Table S4) were added to the cell-RNP suspension. The suspension was transferred to a single 20 μl electroporation cuvette on a 16-well strip (P3 Primary Cell 96-well-Nucleofector Kit, Lonza). Electroporation was carried out on a 4D nucleofector device (Lonza) using programs DI-100 (CB17 HSCs) and EO-100 (C57BL/6 HSCs). Cells were immediately recovered in pre-warmed medium and gently split-transferred into 3 wells on a human fibronectin-coated 24-well plate (Corning) at 1 ml per well. A medium change was performed one day after nucleofection, and further medium changes were performed every 2-3 days.

#### Indel and HDR quantification in bulk-expanded cultures

To quantify indel and HDR rates from bulk cultured cells, genomic DNA (gDNA) was extracted using NucleoSpin Tissue XS columns (Macherey-Nagel). DNA concentration was measured on a Nanodrop spectrophotometer (Thermo Fisher). For murine *Prkdc* editing experiments, 1-10 ng of gDNA was used for polymerase chain reactions (PCR), formulated as 0.5 μM forward and reverse primers (Prkdc_inner_F, Prkdc_inner_R), 10 μl 2X buffer, and 0.5 U of Gflex *Thermococcus* DNA polymerase (Takara) in a 20 μl reaction. The PCR reaction setup was as follows: initial denaturation 94°C, 60s; followed by 35 cycles of denaturation 98°C, 10s; annealing 60°C, 15s; extension 68°C, 45s; and final extension 68°C, 45s. For human *HBB* genotyping, a nested PCR design was devised. Two microliters of gDNA were used with KOD FX Neo polymerase (Toyobo) and primers: HBB_outer_F, HBB_outer_R according to the manufacturer’s instructions. The PCR reaction setup was as follows: initial denaturation 94°C, 120s; followed by 35 cycles of denaturation 98°C, 10s; annealing 60°C, 30s; extension 68°C, 45s; and final extension 68°C, 120s. The second PCR was performed with two microliters of the first reaction and HBB_inner_F, HBB_inner_R, using identical cycle conditions. PCR products were separated on a 1.5% agarose gel via electrophoresis and fragments corresponding to the expected amplification target were cut and gel-purified using the Wizard SV gel and PCR clean-up system (Promega). Fourty nanograms of purified fragment was subjected to Sanger sequencing (FASMAC, Japan) using sequencing primers (Prkdc_inner_F, huHBB_seqprimer). For assessment of HDR rates in bulk cultures, we used the web-based tool Inference of CRISPR edits (ICE, Synthego, https://ice.synthego.com). Sequences from non-edited HSCs were provided as negative control samples. Potential off-target sites associated with the designed *Prkdc* gRNA were identified using COSMID (CRISPR Off-target Sites with Mismatches, Insertions, and Deletions)^49^ with up to 3 mismatches in the absence of indels in the seed sequence and 2 mismatches in the presence of one insert or deletion. All targets showing a score <3 were amplified using the same cycling conditions outlined above. For bulk expansion cultures, only “inner” primer pairs were used for PCRs (see Table S4). Sequencing was performed with either forward or reverse PCR primers (specified with “SEQ” in Table S4), except off-target site #6, for which a dedicated sequencing primer was designed (OT_06_SEQ). On- and off target indel frequency was calculated with the Tracking of Indels by Decomposition (TIDE) algorithm.^47^

#### ddPCR copy number quantification

We used droplet digital PCR to quantify the frequency of large deletions after gene editing in both bulk and single cell murine and human editing models.^16^ In both settings, five primer-probe sets were designed around the cutsite (Figures S1F and S6A; Table S4). Reactions were carried out with ddPCR Supermix for Probes (No dUTP)(Bio-Rad) using 2 μl of gDNA, 225 nM of each primer and 50 nM of each probe in a 20 μl reaction. Murine *Dot1l*^16^ (SUN-labeled, Table S4) and human *RRP30* (Bio-Rad Copy Number Assay (HEX)) were used as internal copy number controls. ddPCR reactions were read out on a QX200 system (Bio-Rad) using the copy number variation method with the control gene copy numbers set to 2. Analysis was performed with QuantaSoft Analysis Pro software (V1.0, Bio-Rad).

#### Flow cytometric analysis of bulk expanded murine HSCs

Cell counting operations were performed on a Countess II cytometer (Thermo Fisher Scientific). For flow cytometric studies of bulk expansion cultures, i.e. HSPCs cultured in 1 ml of expansion media, a 100 μl aliquot was removed from the culture well, washed in PBS, and stained with lineage antibodies (PB- and BV421-conjugated against Gr1[LY-6G/LY-6C], CD11b, CD4, CD8, CD45R[B220], TER119), CD34-FITC, CD201-PE, Sca-1-APC/Cy7, c-Kit-APC, CD150-PE/Cy7 antibodies for 45 minutes. After washing once with PBS, cells were analyzed on a FACSVerse or FACSAria II flow cytometer (BD).

#### Peripheral blood analyses

For chimerism and lineage analysis, peripheral blood was drawn from mice by retro-orbital sinus sampling under general anesthesia. Red blood cells (RBC) in a sample of 40 μl were lysed in 1 ml of Ammonium-Chloride-Potassium (ACK, 0.15 M NH4Cl, 0.01 M KHCO3, 0.1 mM Na2EDTA) buffer for 15 minutes at room temperature. RBC lysis was repeated 2 times. Lysed blood cells were stained with Gr1-PE, CD11b-PE, CD4-APC, CD45R[B220]-APC/eFluor 780, CD8-PE/Cy7 for SCID mouse samples and with Gr1-PE, CD11b-PE, CD4-APC, CD8a-APC, CD45R[B220]-APC/eFluor 780, CD45.1-PE/Cy7 and CD45.2-BV421 for C57BL/6 mice samples for 30 minutes at room temperature. Cells were resuspended in 200 μl PBS/PI before recording events on a FACSVerse (BD) analyzer using the appropriate filters and settings.

#### Fluorescence-activated single cell index sorting

CD34^-^CD150⁺KSL cells were isolated from C57BL/6 mice and cultured on a 96-well dish in PVA-HSC expansion medium at 50 cells per well. After 10 days, cells were stained with antibodies against KSL (biotinylated lineage-antibodies (same mixture as used in ‘Murine HSC isolation’), followed by c-Kit-APC/H7, Sca-1-BV605, and streptavidin-BV421). Cells were then divided into two sets, and each set was stained with an antibody panel (panel 1: CD34-FITC, CD48-APC, CD105-PE; panel 2: CD135-APC, CD201-PE, CD150-PE/Cy7). We cloned single KSL cells using the index sorting function on a FACSAria II (BD). Well location and expression data of the sorted clones were extracted using the IndexSort plugin for FlowJo.^42^ Dimensionality reduction was performed with the UMAP plugin for FlowJo.^43^ Expression data of the sorted clones were log-transformed and normalized to mean.

#### RNA-seq analysis of expanded HSCs

Expanded cells were washed in PBS once and stained with the identical panel specified in ‘Analysis of bulk expanded cells’, except for CD48-FITC, which was used instead of CD34-FITC. Over 5000 cells per population were sorted into 1.5 ml tubes and subsequently lysed in 600 μl Trizol LS reagent (Thermo Fisher Scientific). RNA purification, library preparation and next-generation sequencing was performed by Tsukuba i-Laboratory, LLC. Libraries were prepared using the SMARTer cDNA synthesis kit (Takara) and the high-output kit v2 (Illumina), followed by sequencing on a NextSeq 500 sequencer (Illumina) at 2x 36 paired end reads. Data normalization and comparative analyses were performed with the DESeq2 package in R.^44^ Genes with an adjusted p <0.05 were considered differentially expressed. Enrichment analysis of differentially expressed genes was performed with the gene set enrichment analysis (GSEA) functions in the clusterProfiler package^45^ using molecular signature database (MSigDB) gene ontology biological process (C5 GO:BP) as well as chemical and genetic perturbations (C2:CGP) gene set collections. Heatmaps were generated with the ComplexHeatmap package.^46^

#### Marker profiling of single cell expanded murine HSC clones

To measure HSC marker expression in single cell-derived clonal cultures, 30 μl aliquots of cells were recovered from HSC colonies (cultured in 200 μl), transferred to a 96-well staining plate, washed in PBS, and stained with either PB/BV421- or FITC-conjugated linage antibodies (Gr1[LY-6G/LY-6C], CD11b, CD4, CD8, CD45R[B220], TER119), CD201-PE, Sca-1-APC/Cy7, c-Kit-APC and CD150-PE/Cy7 for 45 minutes at room temperature. After washing with PBS on-plate, cells were resuspended in 200 μl PBS/PI and examined on a FACSVerse analyzer (BD) using appropriate filters and settings. Acquisition time was set to 20 seconds to ensure enough cells remained for genomic DNA extraction, if necessary.

#### Murine TPO ELISA

Ham’s F12 with 100 ng/ml muTPO was supplemented with the indicated polymers and cultured for 3 days at 37°C. After the incubation period, 10 μl of the cultures were diluted tenfold and assayed for TPO concentrations using a commercial ELISA kit (Mouse Thrombopoietin Quantikine ELISA Kit, R&D systems) according to the manufacturer’s instructions.

#### Genotyping of Prkdc-edited single cell HSC clones

To quantify HDR in single cell expanded clones of *Prkdc*-corrected HSCs (genotyping), cells left over from HSC marker profiling (see previous section) were subjected to gDNA extraction using NucleoSpin Tissue XS columns (Macherey-Nagel). gDNA was eluted in 18 μl of ddH_2_O. Only clones containing CD201^+^CD150^+^KL cells were selected for genotyping. For genotyping of the *Prkdc* locus, a nested PCR strategy was employed. The outer PCR formulation was 5 μl of gDNA, 0.5 μM forward and reverse outer primers (Prkdc_outer_F, Prkdc_outer_R) and 12.5 μl of Q5 2X master mix (containing Q5 DNA polymerase, dNTPs and Mg^2+^) (New England Biosciences) in a 25 μl reaction. The PCR reaction setup was as follows: initial denaturation 98°C, 30s; followed by 35 cycles of denaturation 98°C, 10s; annealing 65°C, 15s; extension 72°C, 45s; and final extension 72°C, 120s. The PCR product was diluted 1:20 for the inner PCR reaction. For this reaction, 1 μl of diluted PCR product was combined with 0.5 μM forward and reverse nested primers (Prkdc_inner_F, Prkdc_inner_R) and 25 μl of Q5 2X master mix in a 50 μl reaction. 700 bp PCR products were purified and sequenced as outlined above (‘indel and HDR quantification in bulk-expanded cultures’). A semi-nested PCR strategy was employed for sequencing of off-target edits, first amplifying all sites in a multiplex PCR reaction using outer and inner primers, followed by a second reaction for individual targets using inner primers only. The multiplex PCR reaction contained primers specific to all off-target loci. The formulation was 5 μl of gDNA, 0.25 μM outer and inner primers, 25 μl 2X buffer, and 1.25 U of Gflex *Thermococcus* DNA polymerase (Takara) in a 50 μl reaction. The PCR reaction setup was as follows: initial denaturation 94°C, 60s; followed by 35 cycles of denaturation 98°C, 10s; annealing 60°C, 15s; extension 68°C, 120s; and final extension 68°C, 45s. The PCR product was diluted 1:20 and used for amplification of individual off-target sites. These reactions were formulated as follows: 1 μl of diluted PCR product, 0.25 μM inner primers, 12.5 μl 2X buffer, and 0.625 U of Gflex *Thermococcus* DNA polymerase (Takara) in a 25 μl reaction. The PCR reaction setup was as follows: initial denaturation 94°C, 60s; followed by 35 cycles of denaturation 98°C, 10s; annealing 60°C, 15s; extension 68°C, 120s; and final extension 68°C, 45s. After agarose gel separation, PCR products were purified and sequenced using the inner reverse primers. Sequencing was performed with either forward or reverse inner PCR primers (specified with “SEQ” in Table S4), except off-target site #6, for which a dedicated sequencing primer was used (OT_06_SEQ). Sequence traces were aligned to reference sequences to check for mutations.

#### Murine HSC stem cell transplantation (SCT)

Cells in *Prkdc*-edited bulk HSC cultures were washed, resuspended in 300 μl PBS and divided into 3 aliquots for transplantation into three recipients. Each recipient received 0.5 x10^6^ cells. For experiments comparing PVA and Soluplus expansion conditions, single cell-derived clones were split into several aliquots for SCT into multiple recipients as stated in the main text. *Ptprc*-edited single cell clones were transplanted into a one recipient. For single cell *Prkdc*-corrected clones, candidate clones were selected based on HSC marker and genotyping and combined to a single dose for transplantation into one CB17/SCID recipient. For SCT with *Prkdc*-corrected cells, 0.2 x10^6^ whole bone marrow (WBM) cells from 10 week old male CB17/SCID mice were added to the graft as support to ensure survival immediately after myeloablation. For non-edited and *Ptprc*-edited C57BL/6-derived HSCs, 0.2 x10^6^ WBM competitor cells from C57BL/6 CD45.1^+^/CD45.2^+^ F1 mice were added unless stated otherwise in the main text. CB17/SCID and C57BL/6 mice were lethally irradiated with 2.5 Gy and 9 Gy, respectively, immediately prior to transplantation. Cells were injected via tail vein injection. Secondary bone marrow transplantations were performed by extracting WBM cells from the primary recipient and transplanting 1.0 x10^6^ cells into lethally irradiated secondary recipients.

#### CDR3 spectratyping

The spectratyping protocol originally published by Pannetrier et al. was followed with modifications by Ahmed et al.^26^^,^^50^ Splenocytes were recovered by crushing freshly excised spleens between two glass slides (Matsunami). CD4^+^ lymphocytes were enriched using CD4 magnetic-activated cell sorting (MACS) positive selection according to anufacturer’s intructions (Miltenyi Biotec) and lysed in 300 μl Trizol reagent (Thermo Fisher) per 10^6^ cells. RNA was purified using the Direct-zol RNA Microprep kit (Zymo) and eluted in 15 μl ddH_2_O. 150 ng of RNA was subjected for cDNA synthesis using Superscript IV reverse transcriptase (Thermo Fisher) according to manufacturer’s instructions. 2 μl of cDNA was used per Vβ PCR reaction. The PCR reaction was formulated as: 2 μl of cDNA, 20 pmol TCR constant region (TCR-Cb) and Vβ gene-specific primer (1 μM final concentration) (see Table S4), 10 μl 2X buffer (containing deoxynucleoside triphosphates (dNTPs) and Mg^2+^), and 0.5 U of Gflex DNA polymerase (Takara) in a 20 μl reaction. The PCR reaction setup was as follows: initial denaturation 94°C, 120s; followed by 40 cycles of denaturation 98°C, 10s; annealing 62°C, 30s; extension 68°C, 90s; and final extension 68°C, 600s. 5 μl of PCR product was then used in a runoff reaction including a FAM-labeled TCR-Cb primer. The reaction mix was formulated as 5 μl PCR product, 4 pmol 5’-FAM-labeled TCR constant region primer (TCR-Cb-FAM, 0.2 μM final concentration), 10 μl 2X buffer (containing deoxynucleoside triphosphates (dNTPs) and Mg^2+^), and 0.5 U of Gflex *Thermococcus* DNA polymerase (Takara) in a 20 μl reaction. The PCR reaction setup was as follows: initial denaturation 94°C, 120s; followed by 20 cycles of denaturation 98°C, 10s; annealing 62°C, 30s; extension 68°C, 90s; and final extension 68°C, 300s. Ten microliters of the reaction mix were used for fragment sizing (performed at FASMAC, Japan). Fragment size analysis was performed on the Thermo Fisher Connect platform (https://apps.thermofisher.com/) using the peak scanner application. Relevant peaks were filtered and imported into Prism software (GraphPad) for further analysis and visualization.^51^ For Kolmogorov-Smirnov normality tests, a threshold level of 0.05 was selected to reject the hypothesis that data was normally distributed.

#### Immunization and ELISA assays

Mice at 20 weeks after SCT with *Prkdc*-corrected HSCs were immunized with 100 μg of nitroiodophenyl (NIP)-conjugated OVA (NIPOVA) (Biosearch Technologies) mixed 1:1 with aluminium hydroxide (Invivogen) intraperitoneally (i.p.). Blood samples were collected after 12 and 19 days post-immunization via retro-orbital sinus sampling. Serum was recovered by centrifugation of whole blood for 10 minutes at 5000g and stored at -20°C. For serum antibody detection, high binding 96-well microplates (Thermo Fisher) were pre-coated with NIP_30_-BSA (Biosearch Technologies) at 2 μg/ml concentration overnight. After blocking wells with 1% BSA/PBS solution, 1:5000 dilutions of serum samples were applied to the wells and incubated overnight at 4^o^C. Wells were washed with 0.05% PBS/Tween-20 (PBS-T) followed by incubation with horse radish peroxidase (HRP)-conjugated secondary antibodies against murine IgG and IgM (1:5000 dilution, Southern Biotech) for 2 hours. Enzymatic reaction was initiated by adding 100 μl of 3,3’,5,5’-Tetramethyl -benzidine (TMB) substrate solution (TCI) to each well, followed by termination of the reaction with 100 μl hydrochloric acid 1M (HCl, TCI). Absorbance readings were obtained on a microplate reader at 450 nm (Molecular Devices).

#### Xenograft transplantation assay

Human A549 cells were modified to constitutively express Akaluc, a firefly luciferase derivative with improved bioluminescent activity.^52^ Cultured cells were transduced with a VSV-G pseudotyped lentiviral vector carrying an Akaluc-P2A-mNeonGreen transgene under the control of the human ubiquitin C (UbC) promoter at an MOI of 10. After 14 days, stably transduced cells were selected by sorting mNeonGreen-positive cells on a FACSAria II (BD). Xenograft transplantations were performed by subcutaneously injecting 5 x10^6^ cells in 100 μl of PBS into the flanks of recipient mice. Prior to intravital imaging, the fur above of the injection site was removed with household depilatory cream. After anesthesia, 50 μl of Akalumine-HCl substrate (15 mM, Wako) were injected intraperitoneally and mice were placed in an IVIS in vivo imaging system (PerkinElmer). Images were acquired after 10-15 minutes using appropriate binning (1) and exposure settings.

#### Human HSC culture and HBB CRISPR/Cas9 gene editing

CD34^+^ cord blood HSCs (0.5-1 x10^6^) were thawed and cultured in human HSC bulk expansion medium (see above) at a density of 0.3-0.5 x10^6^ cells/ml before gene editing on day 2. The guide RNA design targeting the human hemoglobin beta gene (HBB_gRNA10) was derived from DeWitt et al. (Table S4).^53^ Editing was performed with the DZ-100 nucleofection program on a 4D nucleofector (Lonza) using the P3 primary cell buffer kit and 20 μl cuvettes (Lonza) analogous to our murine HSC editing protocol with the same amounts of Cas9 protein, sgRNA and ssODN template (synthesized at IDT, Table S4). After editing, cells were allowed to recover in fresh bulk expansion medium overnight before viable cells were cloned on day 3.

#### Human HSC single cell cloning and differentiation-expansion cultures

For single cell HSC expansion, we adopted a previously established protocol for the generation of erythro-myeloid-megakaryocytic colonies from single HSC clones.^54^ Briefly, two days prior to HSC cloning, flat-bottom 96-well plates (Corning) were treated with 50 μl gelatin (0.2%) (Sigma-Aldrich) for 1 hour. After removing the gelatin solution, 1.5 x10^3^ MS-5 murine bone marrow stroma cells in 100 μl HS5100 medium (Stemcell Technologies) were seeded into each well and allowed to become adherent. Two days later, immediately prior to cloning, H5100 medium was replaced with 100 μl of HSC differentiation-expansion medium (StemPro34 (Thermo Fisher) supplemented with 1% PSG and human cytokines FLT3L (20 ng/ml), GM-CSF (20 ng/ml), SCF (100 ng/ml), TPO (100 ng/ml), EPO (3 ng/ml), IL-2 (10 ng/ml), IL-3 (10 ng/ml), IL-6 (50 ng/ml), IL-7 (20 ng/ml) and IL-11 (50 ng/ml); all from Peprotech). Viable (PI negative) HSCs were cloned by fluorescence-activated cell sorting (FACSFusion, (BD)) at a density of one cell per well. Single clones were cultured for 14 days, with the addition of 100 μl fresh medium after seven days. At the end of the culture period, wells containing colonies were marked for downstream analyses (genotyping, ddPCR and transplantation assays).

#### Genotyping of HBB-edited single cell human colonies

Fifty microliters were recovered from each well and transferred to a fresh 96-well plate. Cells were spun down on the plate at 400g for 5 minutes. Using a multichannel pipette, as much supernatant as possible was removed without disturbing the cell pellet. Adopting a protocol outlined in a previous report,^55^ 20 μl of Arcturus PicoPure DNA Extra (Thermo Fisher) extraction solution was added to each well and the crude lysate was incubated at 60°C for 3 hours, followed by 75°C for 30 minutes. As for the Prkdc genotyping protocol, a nested PCR design was devised. For the first genotyping PCR, two microliters of the lysate were used with KOD FX Neo polymerase (Toyobo) and primers: HBB_outer_F, HBB_outer_R according to the manufacturer’s instructions. The PCR reaction setup was as follows: initial denaturation 94°C, 120s; followed by 35 cycles of denaturation 98°C, 10s; annealing 60°C, 30s; extension 68°C, 45s; and final extension 68°C, 120s. The second PCR was performed with two microliters of the first reaction and HBB_inner_F, HBB_inner_R, using identical cycle times. Analogous to our murine *Prkdc* genotyping workflow, PCR products were gel purified and Sanger sequenced (huHBB_seqprimer), and editing outcomes were analyzed with TIDE.

#### Human hematopoietic colony phenotyping

For phenotypic analysis of generated hematopoietic colonies, we followed a previously published protocol by Notta et al.^56^ Briefly, 30 μl of cell culture were transferred from each well onto a fresh 96-well plate. Cells were washed on plate once (400 g, 5 min.) and stained with a lineage marker mix (huCD41-BV421, huCD235a-FITC, huCD71-PE, huCD34-APC, huCD33-PE/Cy7, huCD45-APC/Cy7). After washing once, cells were read out on a FACSVerse analyzer (BD) equipped with a high throughput sampler using the appropriate filters and settings. CD235a expression marked erythroid cells, while CD71 expression and CD33 indicated megakaryocytic and myeloid lineages, respectively. Ten or more positive events were required to consider a colony lineage positive (Figure S6C).

#### Human hematopoietic colony SCT and analysis

Selected human hematopoietic clones were combined and transplanted into 8-10 week-old male NOG mice (In-Vivo Science Inc.) via intrasplenic injection. Mice were irradiated with 1.5 Gy immediately prior to transplantation. Seven to ten days post transplantation, the recipients were sacrificed, and cells were recovered from the spleen and bone marrow. Cells were stained with huCD45-APC/Cy7 and muCD45.1-eFluor 450, and huCD45^+^ cells were sorted on a FACSFusion (BD) cell sorter. Genomic DNA was isolated and HBB editing outcomes quantified as described above (indel and HDR quantification in bulk-expanded cultures).

#### Whole exome sequencing (WES) of single cell-expanded clones

To identify mutations acquired during single cell expansion, we isolated c-Kit^+^ cells from the bone marrow of one C57BL/6 mouse. Genomic DNA was isolated from an aliquot of this population (NucleoSpin Tissue column, Macherey-Nagel). From this parental c-Kit^+^ population, CD34^-^CD150^+^KSL cells were cloned and cultured in HSC expansion medium for 28 days, followed by gDNA extraction.

WES Library generation and sequencing was performed at Macrogen Japan Corp using on a NovaSeq sequencer (Illumina). Target enrichment by was performed using SureSelect (Agilent). FASTQ files were mapped with Burrows-Wheeler Aligner (BWA). PCR duplicates were flagged with Picard. Base Quality Score Recalibration and variant calls were performed with GATK. Variant annotation was performed with SnpEff. Sequence data was annotated using the mouse reference genome (GRCm39) and filtered for mutations that were present only in the expanded clone and not in the parental population. We only considered nonsynonymous mutations with a coverage ≥10.

To detect sequence divergence in a gene-edited, expanded and transplanted clone, CD45.1^+^c-Kit^+^Lin^-^ cells were isolated from secondary recipients and gDNA was extracted for WES analysis. Since the parental population’s data was not available, we compared the exome sequences to the mouse reference genome and searched for divergence in the genes specified in the text.

### Quantification and statistical analysis

Details regarding employed statistical tests as well as number of subjects and groups are stated in the figure legends. Student’s t-tests, one- and two-way analysis of variance (ANOVA) were performed in Prism (version 9.1, Graphpad). Error bars denote standard deviations, unless otherwise stated in the figure legends. Statistical evaluation surrounding RNA-seq analysis, correlation calculations between chimerism and CD201^+^CD150^+^KSL marker expression, as well as select visualizations of peripheral blood lineage distributions were performed in R version 4^57^ with the appropriate packages (outlined in key resources table). Pictograms and illustrations were generated with BioRender (https://www.biorender.com/).

## Data Availability

• The RNA-seq and whole exome sequencing data have been deposited at Gene Expression Omnibus (GEO) and Sequence Read Archive (SRA) and are publicly available as of the date of publication. Accession numbers are listed in the key resources table. Data belonging to figures have been deposited at Mendeley Data and are publicly available as of the date of publication. The DOI is listed in the key resources table. • This paper does not report original code. • Any additional information required to reanalyze the data reported in this paper is available from the lead contact upon request.

## Supplemental information

Document S1. Figures S1–S6, Tables S1–S4, and supplemental references

Document S2. Article plus supplemental information
