# Supplementary material for: Controlling genetic heterogeneity in gene-edited hematopoietic stem cells by single-cell expansion
Source: Cell Stem Cell. 2023 Jul 6;30(7):987–1000.e8. doi: 10.1016/j.stem.2023.06.002 (PMC10338855; doi:10.1016/j.stem.2023.06.002)
Supplement: Document S1. Figures S1–S6, Tables S1–S4, and supplemental references [file mmc1.pdf]

Cell Stem Cell, Volume *30*

Supplemental Information

Controlling genetic heterogeneity in gene-edited hematopoietic stem cells by single-cell expansion

Hans Jiro Becker, Reiko Ishida, Adam C. Wilkinson, Takaharu Kimura, Michelle Sue Jann Lee, Cevayir Coban, Yasunori Ota, Yosuke Tanaka, Meike Roskamp, Tsubasa Sano, Arinobu Tojo, David G. Kent, and Satoshi Yamazaki

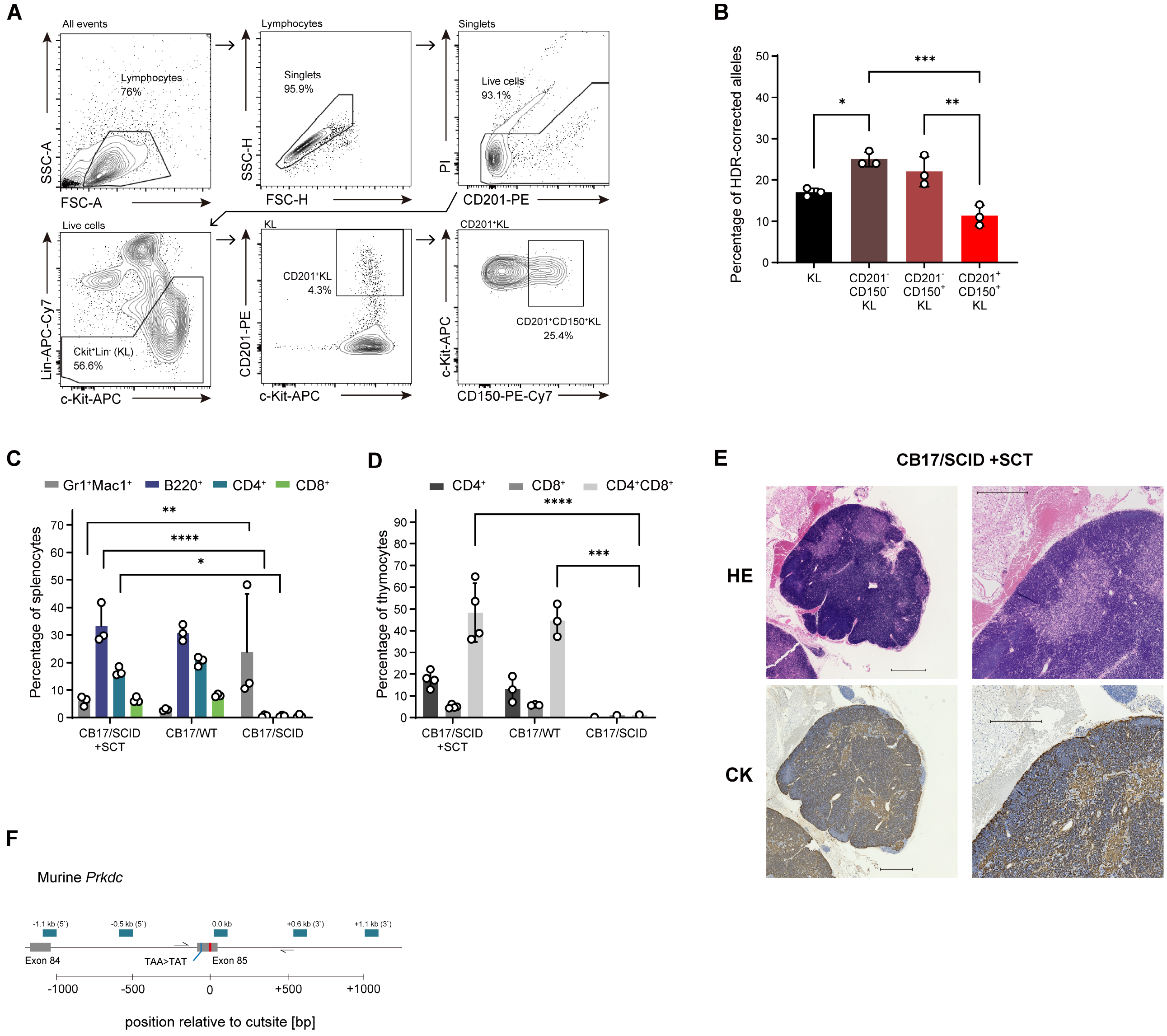

Fig. S1, Becker et al.

Figure S1. Functional correction of *Prkdc*^{scid} HSCs. Related to Fig. 1.

(A) Gating strategy for the isolation of $CD201^{+}CD150^{+}KL$ cells from CB17/SCID mouse BM. **(B)** Frequency of HDR^{+} alleles within phenotypically defined HSPC populations 7 days post gene editing (n=3 cultures). **(C)** Immunophenotype of splenocytes 20 weeks post-SCT (n=3 mice per group). **(D)** Frequencies of double ($CD4^{+}CD8^{+}$) and single positive ($CD4^{+}$ and $CD8^{+}$) thymocytes 20 weeks post-SCT. Data points represent individual mice. **(E)** Sections of thymi isolated from a CB17/SCID recipient transplanted with gene edited HSCs 20 weeks post-SCT. Upper panel: hematoxylin-eosin (HE), lower panel: cytokeratin (CK) stains; Magnified section of left panels (scale bar: 500 µm) shown on right (scale bar: 300 µm). **(F)** Design of ddPCR probes for copy number assays. Blue boxes: probes; red line: cut site; blue line: targeted mutation. Black arrows: PCR primers.

One-(B) and two-way(C, D) ANOVA with Tukey's multiple comparison test.
Error bars represent SD. *P<0.05, **P<0.01, ***P<0.001, ****P<0.0001.

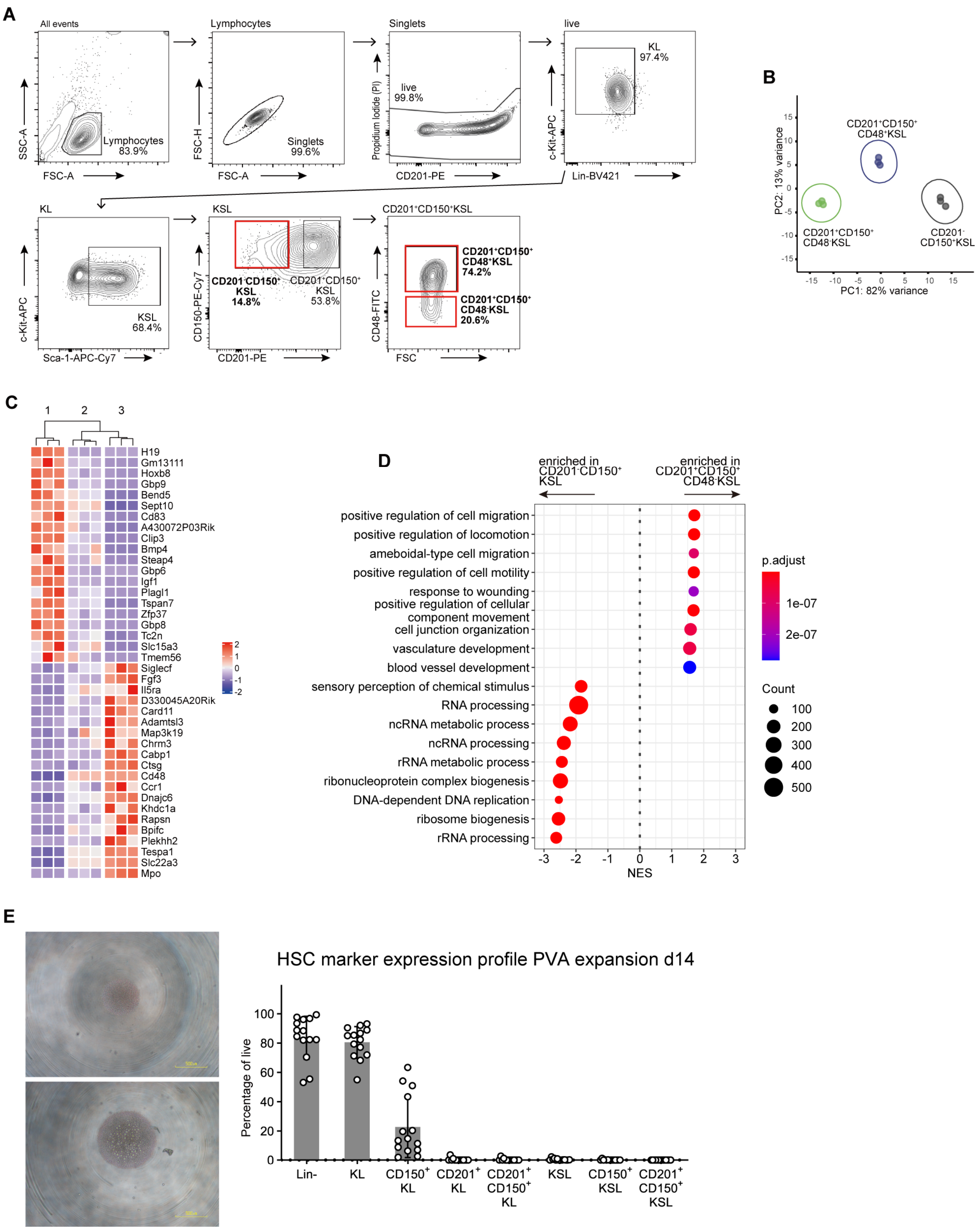

Fig. S2, Becker et al.

Figure S2. Transcriptional and immune phenotype of cultured, gene-edited HSCs. Related to Fig. 2. **(A)** Gating strategy applied for the isolation of $CD201^+CD150^+CD48^-KSL$, $CD201^+CD150^+CD48^+KSL$ and $CD201^-CD150^+KSL$ cells for RNA-seq analysis on day 10 of bulk expansion. **(B)** Principal component analysis (PCA) of $CD201^+CD150^+CD48^-KSL$, $CD201^+CD150^+CD48^+KSL$ and $CD201^-CD150^+KSL$ cells (n=3 replicates). (**C)** Heatmap showing the top 40 differentially regulated (up- and down-regulated) genes in $CD201^+CD150^+CD48^-KSL$ (1) and $CD201^-CD150^+KSL$ (3) populations, with expression data of $CD201^+CD150^+CD48^+KSL$ (2) cells (n=3 replicates). **(D)** Nine most significantly enriched GO terms (biological process) in $CD201^+CD150^+CD48^-$ and $CD201^-CD150^+KSL$ populations. **(E)** Expansion of precultured and cloned $CD201^+CD150^+CD48^-KSL$ cells. $CD34^-CD150^+KSL$ cells were isolated and expanded for 10 days before cloning. The colonies generated from these clones in PVA-based expansion medium were analyzed 14 days post-sort. Left: Representative images of single clone-derived HSC colonies 13 days post-sort. Right: Phenotypic fractions within expanded colonies, as a percentage of live cells (n=14).

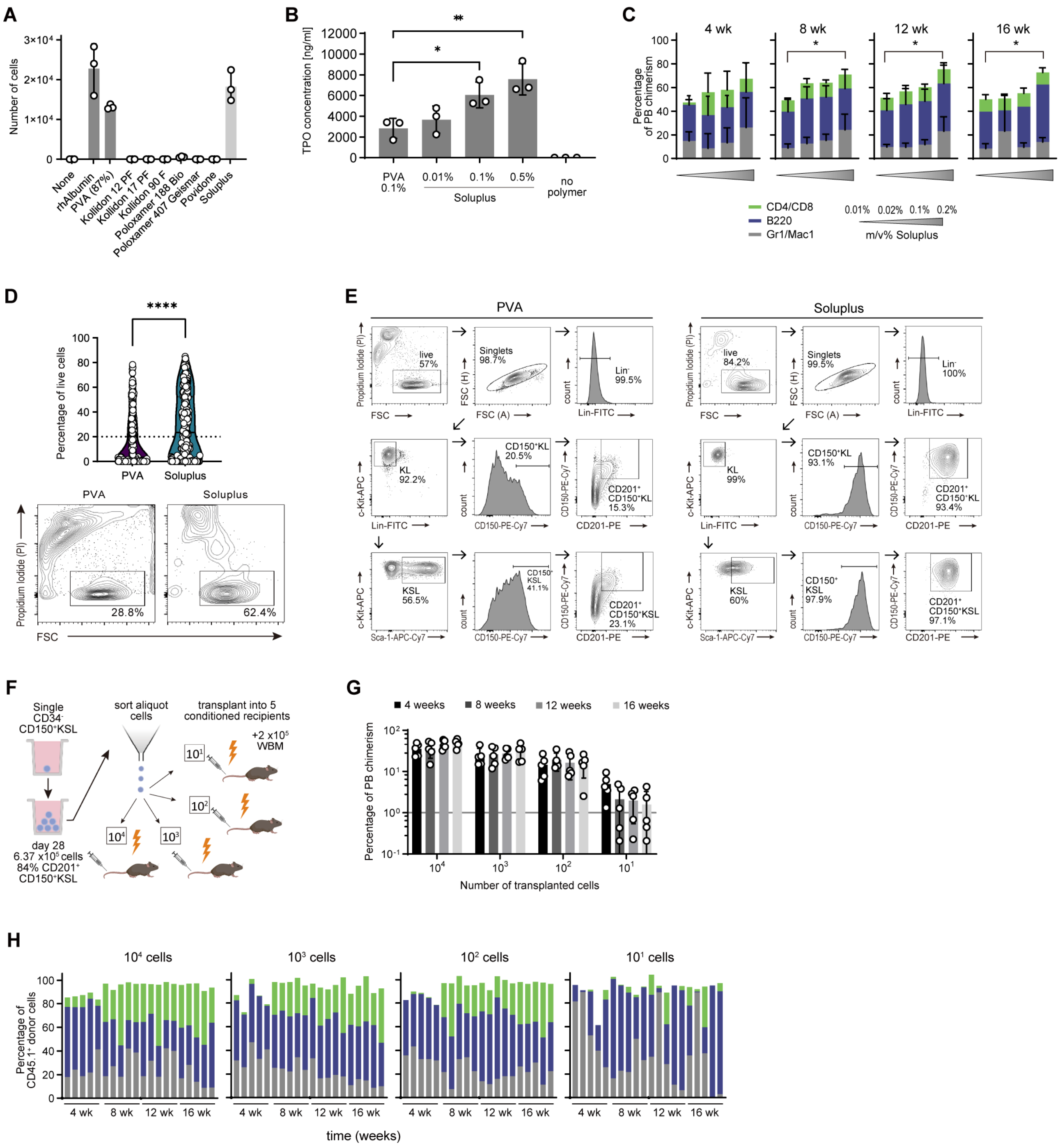

Fig. S3, Becker et al.

Figure S3. Titration of Soluplus supplementation and immune phenotype of Soluplus-expanded HSC clones. Related to Fig. 3.

(A) Albumin replacement polymer screening for *ex vivo* expansion of murine HSCs. 50 freshly isolated C57BL/6 $CD34^-$KSL cells were cultured in media supplemented with the indicated polymers for 7 days. Recombinant human albumin and PVA (87% hydrolyzed) served as positive controls. Concentration of all polymers was 0.1% (m/v). Total cells were counted to assess growth support (n=3 cultures). **(B)** Murine TPO ELISA. Ham's F12 with 100 ng/ml TPO was supplemented with the indicated polymers and cultured for 3 days. ANOVA with Tukey's multiple comparison test. (n=3). **(C)** Soluplus supplementation titration assay. Fifty C57BL/6-Ly5.1 ($CD45.1^+$) $CD34^-CD150^+$KSL cells grown in titrated concentrations of Soluplus (0.01%, 0.02%, 0.1% and 0.2%) were expanded for 14 days and split-transplanted into $CD45.2^+$ recipients (n=4 to 9 per group) against 2 $x10^5$ $CD45.1^+/CD45.2^+$ whole bone marrow competitor cells. Peripheral blood (PB) chimerism and lineage distribution is shown. Supplementation with 0.2% Soluplus produced mild micelle formation in cultures, which was not toxic, but occasionally obstructed the visibility of cells. Two-way ANOVA with Tukey's multiple comparison test. **(D)** Left panel: Percentage of viable cells in HSC colonies grown from freshly isolated single $CD34^-CD150^+$KSL cells after 19 days of culture in PVA (n=288)- and Soluplus (n=290)-based media, as evaluated by flow cytometry (%PI^- of all events). Right panel: Representative FACS plots of single-cell derived colonies (day 19). Two-tailed Mann-Whitney test. **(E)** Representative FACS plots of individual clones expanded in PVA- and Soluplus-supplemented media. **(F)** Schematic of limiting dilution assay (LDA). **(G)** Donor PB chimerism four to 16 weeks post-SCT (5 mice per group). Cutoff level (1%) denoted with gray line. **(H)** PB lineage distribution of $CD45.1^+$ donor cells. Each bar represents an individual recipient.

Error bars represent SD. *P<0.05, **P<0.01, ***P<0.001, ****P<0.0001.

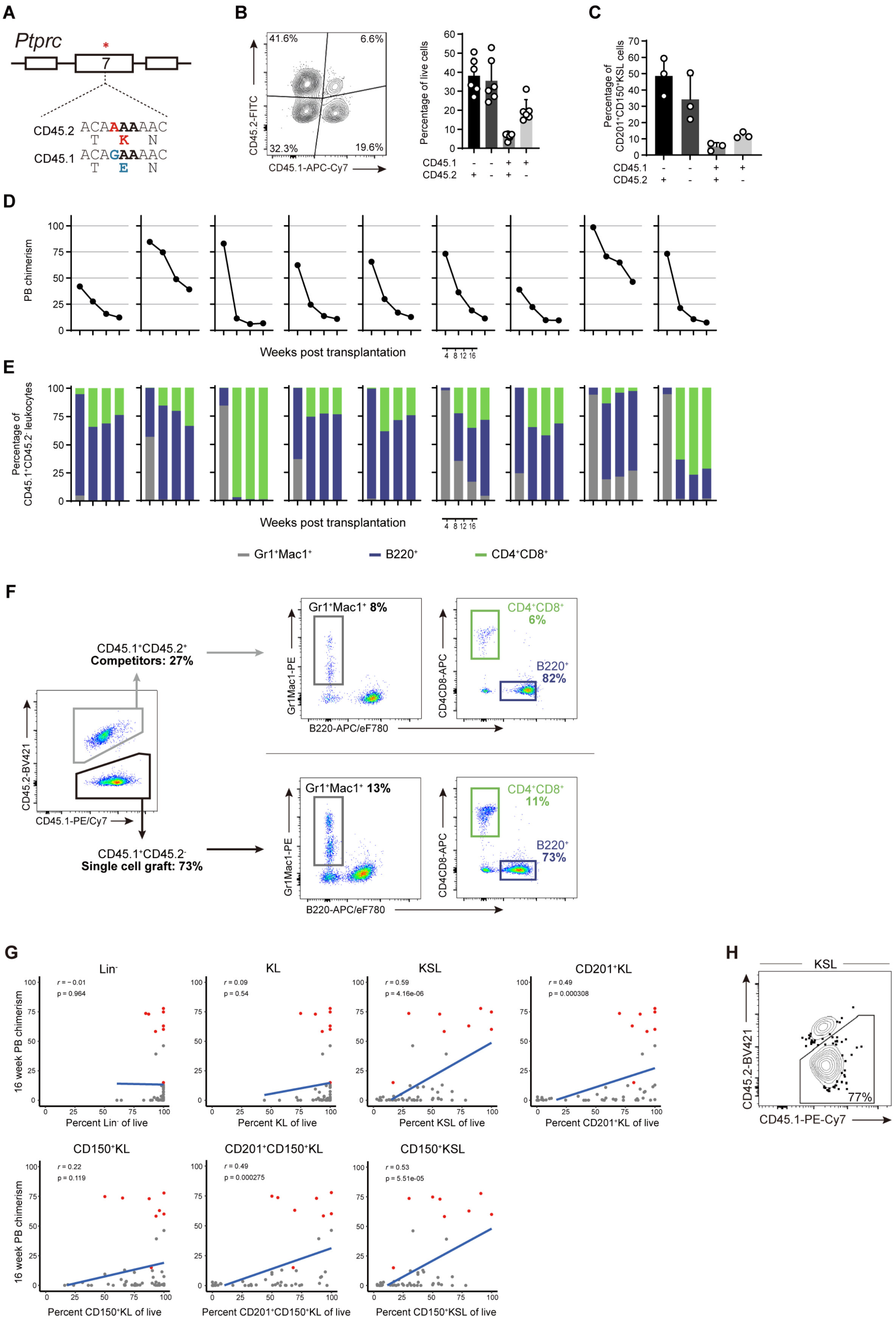

Fig. S4, Becker et al.

Figure S4. *Ptprc* allele conversion efficiency and single clone transplantation assays. Related to Fig. 4. **(A)** Genomic context of the *Ptprc*^{a} and *Ptprc*^{b} alleles. White boxes: exons. *denotes site of SNP. **(B)** Distribution of CD45 phenotypes among live cells 4 days post-editing. Representative FACS plot (left) and summary data (right, n=6 cultures). **(C)** Distribution of CD45 phenotypes among CD201^{+}CD150^{+}KSL cells 4 days post-editing (n=3 cultures).

(D-E) CD45.1^{+} chimerism **(D)** and lineage distribution **(E)** in single recipients that did show LT chimerism ≥5% but did not display multilineage distribution (defined as each lineage ≥5%). Four to 16 weeks post-SCT. Related to Fig. 4d-e. **(F)** Peripheral blood analysis of one of the transplanted mice from Fig. 4D at 16 weeks post-SCT. The single cell graft (CD45.1^{+}CD45.2^{-}) as well as the whole bone marrow competitor graft (CD45.1^{+}CD45.2^{+}) contribute similar amounts to the three lineages examined, suggesting that there is no linage bias in the expanded HSC graft. **(G)** Correlation plots of different expansion culture phenotypes versus LT donor chimerism (16 weeks). Red dots indicate multilineage and LT repopulating clones. Pearson correlation. **(H)** Bone marrow CD45.1^{+} donor chimerism within the KSL compartment of a representative primary recipient.

Error bars represent SD.

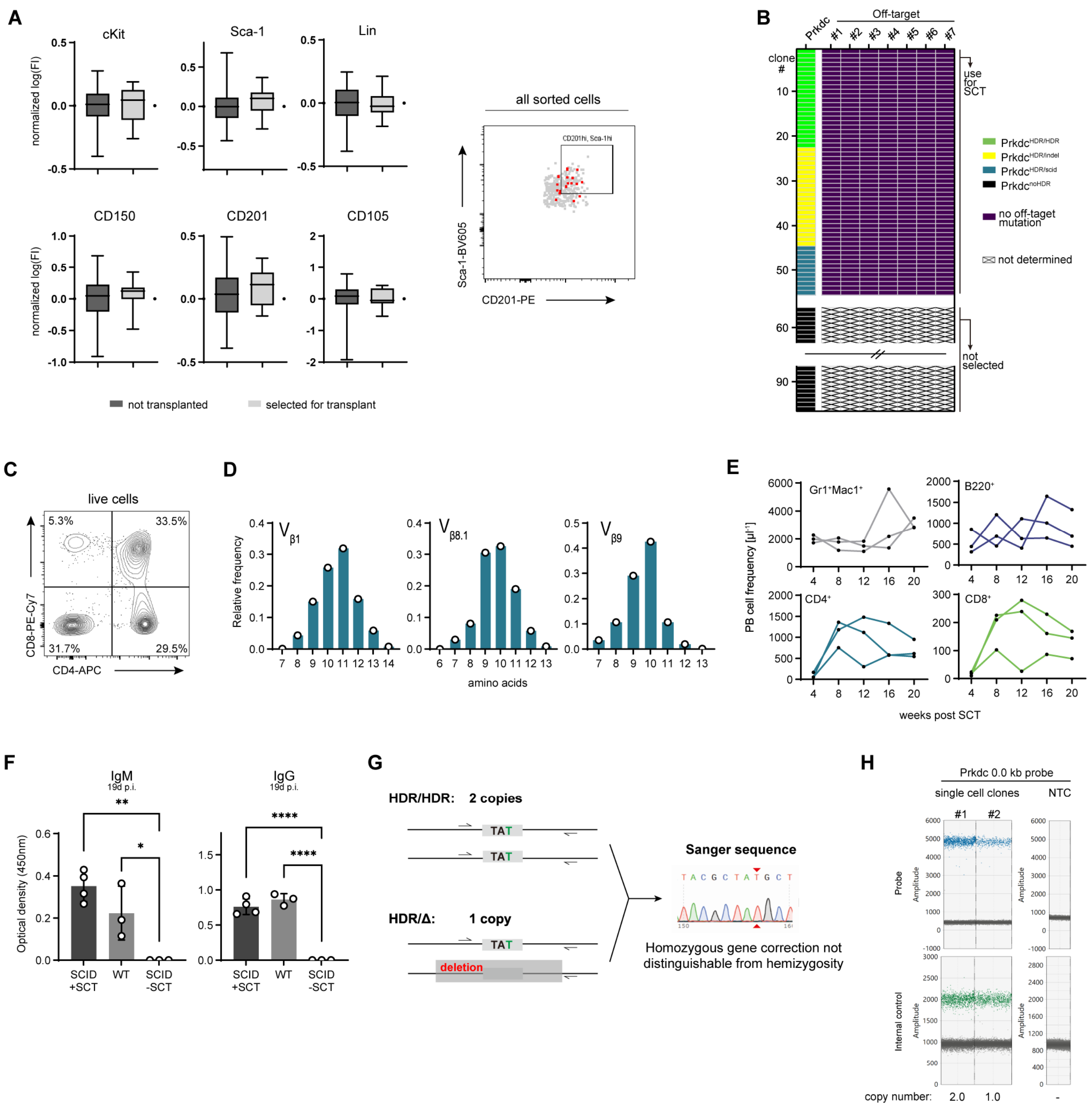

Fig. S5, Becker et al.

Figure S5. Functional rescue of SCID immunodeficiency with edited and single- cell expanded HSC graft. Related to Fig. 5.

(A) Quantification of markers associated with HSC expansion. Marker expression at sorting was compared between clones that were transplanted after the expansion period ($CD201^+CD150^+KL\geq10\%$, HDR^+) and those that were not (remaining clones). Left: Fluorescence intensity (FI) measured at sorting. Data presented as log-transformed and normalized to mean. Box plots with whiskers showing minimum and maximum. Right: Representative FACS sorting plot, transplanted clones indicated in red. **(B)** Genotypes of all profiled candidate clones (n=96). **(C)** Representative FACS plot showing frequencies of double ($CD4^+CD8^+$) and single positive ($CD4^+$ and $CD8^+$) cells from the thymus of a CB17/SCID recipient 20 weeks after transplantation. **(D)** CDR3 length spectratype analysis of the Tcrb-V1, -V8.1 and -V9 genes in splenic $CD4^+$ cells. Each bar represents the relative frequency of a CDR3 length species (n=1 mouse). **(E)** Absolute PB cell frequencies in CB17/SCID recipients. (F) Serum levels of NIP_{30}-specific IgG and IgM 19 days after immunization (CB17/SCID+SCT: n=4 mice; CB17/WT and treatment-naïve CB17/SCID: n=3). (G) LD schematic **(H)** Example data showing ddPCR quantification of *Prkdc* alleles (0.0kb probe) in two clones.

One-(E) and two-way (A, B) ANOVA with Tukey's multiple comparison test.
Error bars represent SD. *P<0.05, **P<0.01, ***P<0.001, ****P<0.0001.

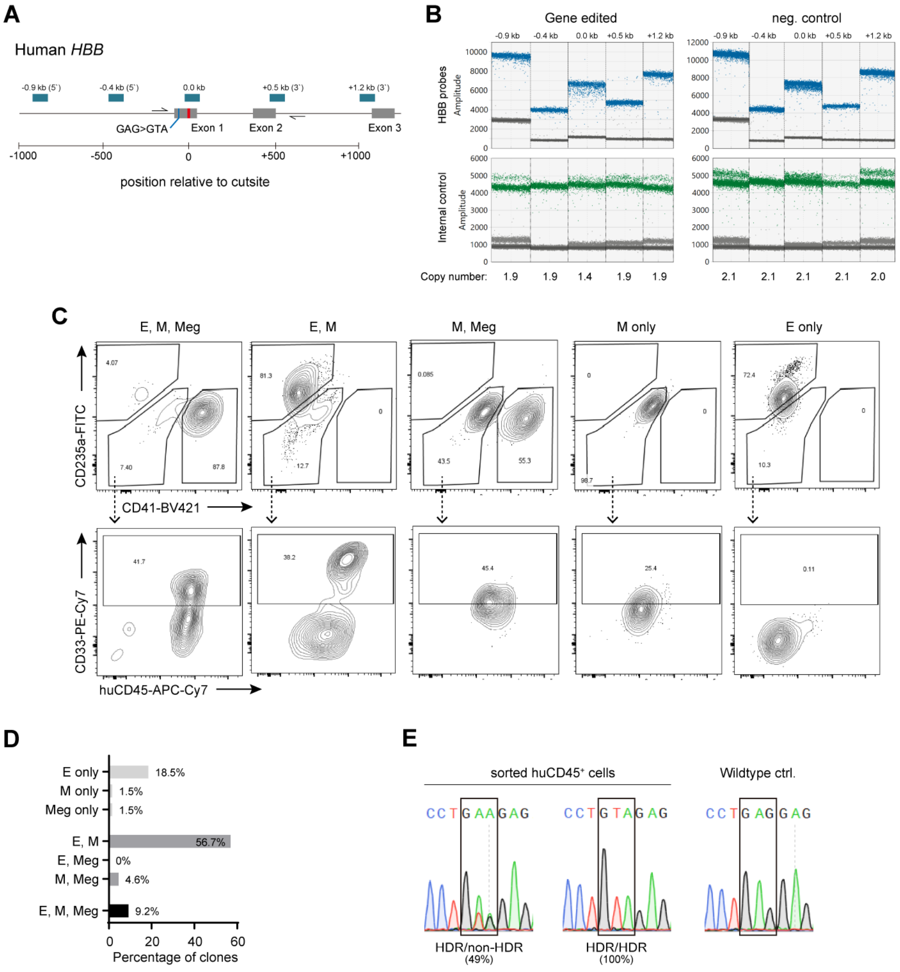

Fig. S6, Becker et al.

Figure S6. Expansion and profiling of single, *HBB* gene-edited human HSCs. Related to Fig. 6.

(A) Editing strategy of the *HBB* locus with ddPCR probes for copy number assays. Blue boxes: ddPCR probes; red line: cut site; blue line: targeted mutation. **(B)** Example ddPCR quantification data of bulk gene edited CB HSCs (left) and negative control (unedited) cells. **(C)** Example FACS plots showing colony differentiation outcomes for erythroid (E, $CD235a^{+}$), myeloid (M, $CD33^{+}$) and megakaryocytic (Meg,$CD41^{+}$)-containing colonies. A lineage was defined positive if more than ten events were detected in the in the relevant gate. **(D)** Summary of single huHSPC differentiation outcomes. E, erythroid; M, Myeloid; Meg, megakaryocytic. (n=65). **(E)** Example sequencing traces from sorted $huCD45^{+}$ cells isolate from NOG recipients transplanted with HPSCs heterozygous ($HBB^{\text{HDR/non-HDR}}$) or homozygous ($HBB^{\text{HDR/HDR}}$) for the SCD mutation. Healthy control showing wild-type sequence (right).

#	Hit	Permutation	Mismatches	Position (GRCm38)	strand	Gene
	CTTACCAAGTTATAACAGCTNGG	-	-	-	-	Prkdc_gRNA1
**1**	ATTACTTAGTTATAACAGCTGGG	No indel	3	Chr5:57647418-57647440	-	4932441J04Rik
**2**	CTTAAAAAGTCATAACAGCTGGG	No indel	3	Chr5:21558299-21558321	-	Fbxl13; Lrrc17
**3**	CTTA-TAACTTATAACAGCTAGG	Del 15, or Del 16	2	Chr14:96347342-96347363	-	Klhl1
**4**	CTTAACAA-TTCTAACAGCTGGG	Del 12	2	Chr8:73726599-73726620	+	No known gene
**5**	CTTAGCAA-TTTTAACAGCTCGG	Del 12	2	Chr1:24130829-24130850	-	No known gene
**6**	CTTCCCAAG-TAGAACAGCTGGG	Del 10, or Del 11	2	Chr4:54112620-54112641	+	No known gene
**7**	CTTCCCAAG-TATTACAGCTTGG	Del 10, or Del 11	2	Chr2:140296967-140296988	+	Sel1l2

Supplemental Table S1. Candidate off-target sites. Related to Fig. 1.

Candidate off-target sites of the guide RNA targeting *Prkdc* were identified using the COSMID algorithm. Mismatches and permutations in the seed region are indicated in red.

The first row shows the seed sequence and PAM of the sgRNA used (Prkdc_gRNA1).

Chr	Position	Reference	Allele	Count	Coverage	Freq.	Control count	Control coverage	Transcript affected	AA change in longest transcript	Gene
**Indels**											
chr1	85504851	CACACACACACACACACACACACACACAG	C	11	39	28.2%	0	13	ENSMUST00000178024.2; c.257_284delCACACACACACACACACACACACACAGA	p.Thr86fs	ENSMUSG00000094127; G530012D18Rik
chr1	85504943	GAC	G	34	101	33.7%	0	39	ENSMUST00000178024.2; c.349_350delCA	p.Gln117fs	ENSMUSG00000094127; G530012D18Rik
chr2	98497361	CAAGAAAACTGAAAATCA	C	75	111	67.6%	0	449	ENSMUST00000099683.2; c.239_255delTGATTTTCAGTTTTCTT	p.Leu80fs	ENSMUSG00000075014; Gm10800
chr4	126059360	TC	T	6	34	17.6%	0	20	ENSMUST00000080919.12; c.2684delG	p.Arg895fs	ENSMUSG00000043962; Thrap3
chr9	3002239	A	ATTTCTCATTTTTCACAT	2	16	12.5%	0	18	ENSMUST00000151376.3; c.575_576insATTTTCTCATTTTTCAC	p.Phe194fs	ENSMUSG00000091028; Gm10722
chr9	3025119	C	CA	2	15	13.3%	0	11	ENSMUST00000099046.4; c.583dupA	p.Ser195fs	ENSMUSG00000095186; Gm10718
chr17	23530080	TC	T	56	355	15.8%	0	93	ENSMUST00000168033.3; c.325delG	p.Glu109fs	ENSMUSG00000091945; Vmn2r114
chr17	23530164	GA	G	184	300	61.3%	0	93	ENSMUST00000168033.3; c.241delT	p.Ser81fs	ENSMUSG00000091945; Vmn2r114
**SNPs**											
chr1	85504886	A	G	15	46	32.6%	0	18	ENSMUST00000178024.2; c.290A>G	p.Glu97Gly	ENSMUSG00000094127; G530012D18Rik
chr1	85504923	G	C	33	78	42.3%	0	36	ENSMUST00000178024.2; c.327G>C	p.Glu109Asp	ENSMUSG00000094127; G530012D18Rik
chr1	85504941	G	C	34	93	36.6%	0	39	ENSMUST00000178024.2; c.345G>C	p.Glu115Asp	ENSMUSG00000094127; G530012D18Rik
chr1	85504970	C	T	43	124	34.7%	0	39	ENSMUST00000178024.2; c.374C>T	p.Ala125Val	ENSMUSG00000094127; G530012D18Rik
chr1	85504990	A	G	42	125	33.6%	0	39	ENSMUST00000178024.2; c.394A>G	p.Ile132Val	ENSMUSG00000094127; G530012D18Rik
chr1	133284294	G	A	23	91	25.3%	0	63	ENSMUST00000094556.3; c.658G>A	p.Glu220Lys	ENSMUSG00000070645; Ren1
chr2	98492825	T	G	11	52	21.2%	0	23	ENSMUST00000099684.4; c.244T>G	p.Cys82Gly	ENSMUSG00000075015; Gm10801
chr2	98497422	T	G	1009	1036	97.4%	0	2036	ENSMUST00000099683.2; c.220A>C	p.Ser74Arg	ENSMUSG00000075014; Gm10800
chr3	98334623	G	T	2	11	18.2%	0	12	ENSMUST00000056096.15; c.387G>T	p.Glu129Asp	ENSMUSG00000050064; Zfp697
chr4	126059371	C	T	6	34	17.6%	0	27	ENSMUST00000080919.12; c.2674G>A	p.Gly892Ser	ENSMUSG00000043962; Thrap3
chr4	126059400	C	T	4	23	17.4%	0	23	ENSMUST00000080919.12; c.2645G>A	p.Arg882Gln	ENSMUSG00000043962; Thrap3
chr7	11775508	T	G	4	18	22.2%	0	12	ENSMUST00000227320.2; c.283T>G	p.Cys95Gly	ENSMUSG00000095864; Vmn1r77
chr7	11775524	C	T	7	18	38.9%	0	13	ENSMUST00000227320.2; c.299C>T	p.Ala100Val	ENSMUSG00000095864; Vmn1r77
chr7	11775731	A	T	4	23	17.4%	0	23	ENSMUST00000227320.2; c.506A>T	p.Tyr169Phe	ENSMUSG00000095864; Vmn1r77
chr9	3000927	C	A	2	11	18.2%	0	12	ENSMUST00000151376.3; c.6C>A	p.Cys2*	ENSMUSG00000091028; Gm10722
chr9	3006907	A	C	3	15	20.0%	0	11	ENSMUST00000179881.2; c.631A>C	p.Asn211His	ENSMUSG00000096385; Gm11168
chr9	3025133	C	T	2	14	14.3%	0	11	ENSMUST00000099046.4; c.596C>T	p.Ser199Leu	ENSMUSG00000095186; Gm10718

chr11	115895814	C	A	4	16	25.0%	0	11	ENSMUST00000106458.2; c.4199C>A	p.Pro1400Gln	ENSMUSG00000020758; Itgb4
chr15	71335125	G	T	16	97	16.5%	0	37	ENSMUST00000022953.10; c.2068C>A	p.Pro690Thr	ENSMUSG00000036800; Fam135b
chr17	23530099	T	C	236	352	67.0%	0	93	ENSMUST00000168033.3; c.307A>G	p.Lys103Glu	ENSMUSG00000091945; Vmn2r114
chr17	23530105	T	G	230	346	66.5%	0	93	ENSMUST00000168033.3; c.301A>C	p.Ile101Leu	ENSMUSG00000091945; Vmn2r114
chr17	23530113	C	A	226	348	64.9%	0	93	ENSMUST00000168033.3; c.293G>T	p.Arg98Ile	ENSMUSG00000091945; Vmn2r114
chr17	23530117	C	G	221	344	64.2%	0	93	ENSMUST00000168033.3; c.289G>C	p.Val97Leu	ENSMUSG00000091945; Vmn2r114
chr17	23530182	A	G	155	269	57.6%	0	93	ENSMUST00000168033.3; c.224T>C	p.Val75Ala	ENSMUSG00000091945; Vmn2r114
chr17	23530186	T	A	153	264	58.0%	0	93	ENSMUST00000168033.3; c.220A>T	p.Asn74Tyr	ENSMUSG00000091945; Vmn2r114
chr17	23530192	A	G	142	249	57.0%	0	93	ENSMUST00000168033.3; c.214T>C	p.Tyr72His	ENSMUSG00000091945; Vmn2r114
chr17	45879194	G	A	7	61	11.5%	0	47	ENSMUST00000024739.14; c.2026C>T	p.His676Tyr	ENSMUSG00000023944; Hsp90ab1
chr17	45879196	G	A	7	59	11.9%	0	48	ENSMUST00000024739.14; c.2024C>T	p.Thr675Ile	ENSMUSG00000023944; Hsp90ab1
chr17	45879205	T	A	7	56	12.5%	0	46	ENSMUST00000024739.14; c.2015A>T	p.Asp672Val	ENSMUSG00000023944; Hsp90ab1

Supplemental Table S2. Mutations in a single cell-derived HSC colony. Related to Fig. 3.

Nonsynonymous mutations (indels and single nucleotide variations (SNPs)) detected in an HSC clone expanded over a period of 28 days. Whole exome sequencing (WES) was performed on the expanded clone and the parent population from which the clone was derived. Both samples were compared to the murine reference genome, and only mutations detected in the expanded clone and not in the parent population were considered.

Chromosome	Position	Reference	Allele	Count	Coverage	Frequency	Transcript affected	AA change in longest transcript	Gene
chr5	147293801	AT	A	38	40	95.0%	ENSMUST00000049324.13; c.1313-40delA	None (intron_variant)	ENSMUSG00000042817; FLT3
chr5	147294645	CT	C	6	18	33.3%	ENSMUST00000049324.13; c.1312+112delA	None (intron_variant)	ENSMUSG00000042817; FLT3
chr1	65198550	TA	T	16	36	44.4%	ENSMUST00000097709.11; c.*73delT	None (3_prime_UTR_variant)	ENSMUSG00000025950; IDH1
chr7	79745297	TG	T	6	34	17.6%	ENSMUST00000107384.10; c.1271+71delC	None (intron_variant)	ENSMUSG00000030541; IDH2
chr7	79748080	CCCAGGG	C	24	24	100.0%	ENSMUST00000107384.10; c.679-66_679-61delCCCTGG	None (intron_variant)	ENSMUSG00000030541; IDH2

Supplemental Table S3. Screen for sequence variants in critical genes in a gene edited and transplanted HSC clone. Related to Fig. 4.

Gene-edited CD45.1$^+$cKit$^+$Lin$^-$ cells were recovered from the bone marrow of CD45.2$^+$ secondary recipients of a single cell-derived HSC graft, followed by WES analysis. We checked several known tumor-associated genes for sequence variants: TP53, FLT3, NPM1, IDH1, IDH2, EVI1, TET2, RUNX1, EZH2 and DNMT3A. Since the parent population was not available, sequences were compared to the mouse reference genome. Therefore, it is possible that some of these variants are part of the genomic background of the mouse strain and have not been acquired during the editing and single cell expansion process.

Supplemental Table 4. Oligonucleotides used in this study. Related to STAR Methods.

Sequences of DNA and RNA oligos used in this work.

**Sequence**	**Source**	**Identifier**
**Synthetic guide RNA (sgRNA) (mN*: Phosphorothioated 2'-O-methyl RNA base)**		
mC*mU*mU*ACCAAGUUAUAACAGCUGUUUUAGAGCUAGAAAUAGCAAGUUAAAAUAAGGCUAGUCCGUUAUCAACUUGAAAAAGUGGCACCGAGUCGGUGCmU*mU*mU*U	This study	Prkdc_gRNA1
mA*mU*mA*CUUCAAUUUGUUUGGAGGUUUUAGAGCUAGAAAUAGCAAGUUAAAAUAAGGCUAGUCCGUUAUCAACUUGAAAAAGUGGCACCGAGUCGGUGCmU*mU*mU*U	This study	Ptprc_gRNA1
mU*mG*mC*CCCACAGGGCAGUAAGUUUUAGAGCUAGAAAUAGCAAGUUAAAAUAAGGCUAGUCCGUUAUCAACUUGAAAAAGUGGCACCGAGUCGGUGCmU*mU*mU*U	DeWitt et al., 2016 [S1]	HBB_gRNA10
**Single-strand oligodeoxynucleotide (ssODN) HDR templates (N*: Phosphorothioated DNA base)**		
G*G*A*TTCAAGAAATAAATGTAACGGAAAAGAATTGGTATCCACAACATAAAATACGCTATGCTAAGAGGAAGTTAGCAGGTGCCAATCCAGCTGTTATAACTTGGTAAGACTTGTGAATGCAGAA*T*C*A	This study	Prkdc_HDR_ssODN_asym
T*G*C*CCAGCATCGTACCTGGCTCACAGTGGAGTACATATGAAATATTGTCACTGTTGCATTTTCTGAAATCAAGGTTTTCTGTCTTCCATTCCAAACAAATGGAAGTATTAGCCTTTTCTTTTGG*T*G*T	This study	Ptprc_HDR_ssODN_asym
T*C*AGGGCAGAGCCATCTATTGCTTACATTTGCTTCTGACACAACTGTGTTCACTAGCAACCTCAAACAGACACCATGGTGCACCTGACTCCTGTAGAGAAGTCTGCGGTTACTGCCCTGTGGGGCAAGGTGAACGTGGATGAAGTTGGTGGTGAGGCCCTGGGCAG*G*T	This study	HBB_HDR_ssODN_asym
**PCR and sequencing primers (FAM-: 5' 6-carboxyfluorescein label)**		
CTTTGTTTTAGGGTCATTACTTGGT	This study	Prkdc_inner_F
TGCTCAGAACTGAAGTCTAAGGT	This study	Prkdc_inner_R
CAATTATCCAGACTATCCCCGAAA	This study	Prkdc_outer_F
TCTTGCCTACACCCTGTAAAGC	This study	Prkdc_outer_R
TCCCTCCTAGAAGCACTTGTT	This study	Ptprc-e7-F
CCCCTAGCGAAATCTCCTGC	This study	Ptprc-e7-R
AGCGGAAGGGCAACTTTACTA	This study	OT_01_outer_F
GATCTCATACACAGACAGGGAAG	This study	OT_01_inner_F
GCATACGCCATTCTGCTCAC	This study	OT_01_inner_R_SEQ[a]
TGCCTGTATGGTAAGCACCC	This study	OT_02_inner_F_SEQ[a]
TTAGTGGCAGGCATGCTTCA	This study	OT_02_outer_R
AGTCACCATTCGTGTGTCCC	This study	OT_02_inner_R
TGGATCTTGTTCATCTGGGGC	This study	OT_03_inner_F_SEQ[a]
GCCTGAGGGACTCAGTATTGT	This study	OT_03_outer_R
AGATTTCAAGATGTCCTTACGA	This study	OT_03_inner_R
TATTAGCACCTACACCAATGCT	This study	OT_04_outer_F
GTGGCACAAAGAAAGATGTATGG	This study	OT_04_inner_F
TGCAGTCCTTGTAAGAGGGT	This study	OT_04_inner_R_SEQ[a]
GATGCAGCTGAGAGACTCGT	This study	OT_05_inner_F_SEQ[a]

CACTCCCTGTGTGTTTGTTTC	This study	OT_05_inner_R
AGTAGGTCTTTGTAGGCACGC	This study	OT_05_outer_R
CGACAACACTCTGACTCCCATA	This study	OT_06_outer_F
CCTTTCCCTTGGGTACTTCTTG	This study	OT_06_inner_F
TGCAAATGACCGGAAATCTGTAAA	This study	OT_06_inner_R
GCCCTCAAGATTTTGCTGTCAAG	This study	OT_06_SEQ[a]
TACCTTCTCACAAGCAGGGAGG	This study	OT_07_outer_F
GAGGAGCCTTATGGAAGAGTTG	This study	OT_07_inner_F
TTAAGGCATTCCTGTCTGCCA	This study	OT_07_inner_R_SEQ[a]
CTGAATGCCCAGACAGCTCCAAGC	Ahmed et al., 2009 [S2]	TCR-Vb1
CATTACTCATATGTCGCTGAC	Ahmed et al., 2009 [S2]	TCR-Vb8.1
TGCTGGCAACCTTCGAATAGGA	Ahmed et al., 2009 [S2]	TCR-Vb8.3
TCTCTCTACATTGGCTCTGCAGGC	Ahmed et al., 2009 [S2]	TCR-Vb9
CTTGGGTGGAGTCACATTTCT	Ahmed et al., 2009 [S2]	TCR-Cb
FAM-CTTGGGTGGAGTCACATTTCT	Ahmed et al., 2009 [S2]	TCR-Cb-FAM
AACTTGGTAAGACTTGTGAATGC	This study	Prkdc_dd_00-2_F
ACACAGTGAAGTGCCATACT	This study	Prkdc_dd_00-2_R
GAAGAGGGAGCACCTGAATTA	This study	Prkdc_dd_5p-500-1_F
TGTTCTTACAGAGGACCAACC	This study	Prkdc_dd_5p-500-1_R
TCAGTTCTGAGCAGTAGTAAGATG	This study	Prkdc_dd_3p-500-1_F
ATTGATTTCTGCAGCTCATTCTC	This study	Prkdc_dd_3p-500-1_R
GCTCACTGACACCATGGAAA	This study	Prkdc_dd_5p-1000-1_F
TCACAATCCAAAGGAGACATGA	This study	Prkdc_dd_5p-1000-1_R
TGGTTCTTCCACCTCCAATC	This study	Prkdc_dd_3p-1000-3_F
CATGATGCCTATGTCTGAGGAG	This study	Prkdc_dd_3p-1000-3_R
GCCCCAGCACGACCATT	This study	Dot1l_dd_1_F
TAGTTGGCATCCTTATGCTTCATC	This study	Dot1l_dd_1_R
AAGTAACTAATGCACAGAGCACAT	This study	huHBB_outer_F
AATGTACTAGGCAGACTGTGTAA	This study	huHBB_outer_R
ATGCTTAGAACCGAGGTAGAGTT	This study	huHBB_inner_F
CCTGAGACTTCCACACTGATGC	This study	huHBB_inner_R
ACCATGGTGCATCTGACTC	This study	huHBB_00-3_F
GGTCTCCTTAAACCTGTCTTGT	This study	huHBB_00-3_R
AACGATCTTCAATATGCTTACCAAG	This study	huHBB_5p-500-3_F
CCCATACCATCAGTACAAATTGC	This study	huHBB_5p-500-3_R
CACGTGGATCCTGAGAACTT	This study	huHBB_3p-500-1_F
CTTCCTATGACATGAACTTAACCATAG	This study	huHBB_3p-500-1_R
GTCCAGGCAGAAACAGTTAGA	This study	huHBB_5p-1000-3_F
CAACCCAAAGTGTGACTATCAATG	This study	huHBB_5p-1000-3_R

ACTGATGTAAGAGGTTTCATATTGC	This study	huHBB_3p-1000-1_F
GCCTAGCTTGGACTCAGAATAA	This study	huHBB_3p-1000-1_R
CTAGGGTTGGCCAATCTACTCC	This study	huHBB_seqprimer
[a]SEQ denoted off-target PCR primers were also used as sequencing primers		
**ddPCR probes (synthesized at IDT)**		
CCAGCTCTCAAGTCG	This study	Dot1l-1_probe (SUN)
TGCAAAGCACTTCACACACTTCTGAGC	This study	Prkdc_00-2_probe (FAM)
ACCAATGTTTCTGGGACCTGAATGCT	This study	Prkdc_5p-500-1_probe (FAM)
AGTGTCAGATCTATACAGTGACTGGTGCT	This study	Prkdc_3p-500-1_probe (FAM)
TGTGAAGGAACCCTCCTTTGATTGGAAGG	This study	Prkdc_5p-1000-1_probe (FAM)
TCTGTACCTCCGGTTCTAGGGAACCT	This study	Prkdc_3p-1000-3_probe (FAM)
CAAGGTGAACGTGGATGAAGTTGG	This study	huHBB_00-3_probe (FAM)
CGTAAATACACTTGCAAAGGAGGATGT	This study	huHBB_5p-500-3_probe (FAM)
AGGGTGAGTCTATGGGACGCTTGA	This study	huHBB_3p-500-1_probe (FAM)
ACCTCCTATTTGACACCACTGATTACCC	This study	huHBB_5p-1000-3_probe (FAM)
CAGCTACAATCCAGCTACCATTCTGCT	This study	huHBB_3p-1000-1_probe (FAM)

Supplemental References

S1. DeWitt, M.A., Magis, W., Bray, N.L., Wang, T., Berman, J.R., Urbinati, F., Heo, S.-J., Mitros, T., Munoz, D.P., Boffelli, D., et al. (2016). Selection-free genome editing of the sickle mutation in human adult hematopoietic stem/progenitor cells. Sci Transl Med *8*, 360ra134-360ra134. 10.1126/scitranslmed.aaf9336.

S2. Ahmed, M., Lanzer, K.G., Yager, E.J., Adams, P.S., Johnson, L.L., and Blackman, M.A. (2009). Clonal Expansions and Loss of Receptor Diversity in the Naive CD8 T Cell Repertoire of Aged Mice. The Journal of Immunology *182*, 784–792. 10.4049/jimmunol.182.2.784.
